# BTR: training asynchronous Boolean models using single-cell expression data

**DOI:** 10.1186/s12859-016-1235-y

**Published:** 2016-09-06

**Authors:** Chee Yee Lim, Huange Wang, Steven Woodhouse, Nir Piterman, Lorenz Wernisch, Jasmin Fisher, Berthold Göttgens

**Affiliations:** 1 Department of Haematology, Wellcome Trust and MRC Cambridge Stem Cell Institute, Cambridge Institute for Medical Research, University of Cambridge, Hills Road, Cambridge, CB2 0XY UK; 2 Department of Computer Science, University of Leicester, Leicester, UK; 3 Biostatistics Unit, Medical Research Council, Cambridge, UK; 4 Microsoft Research Cambridge, Cambridge, UK; 5 Department of Biochemistry, University of Cambridge, Cambridge, UK

**Keywords:** Asynchronous Boolean model, Single-cell gene expression, Model learning, Network reconstruction, BOOLEAN scoring function, Executable model

## Abstract

**Background:**

Rapid technological innovation for the generation of single-cell genomics data presents new challenges and opportunities for bioinformatics analysis. One such area lies in the development of new ways to train gene regulatory networks. The use of single-cell expression profiling technique allows the profiling of the expression states of hundreds of cells, but these expression states are typically noisier due to the presence of technical artefacts such as drop-outs. While many algorithms exist to infer a gene regulatory network, very few of them are able to harness the extra expression states present in single-cell expression data without getting adversely affected by the substantial technical noise present.

**Results:**

Here we introduce BTR, an algorithm for training asynchronous Boolean models with single-cell expression data using a novel Boolean state space scoring function. BTR is capable of refining existing Boolean models and reconstructing new Boolean models by improving the match between model prediction and expression data. We demonstrate that the Boolean scoring function performed favourably against the BIC scoring function for Bayesian networks. In addition, we show that BTR outperforms many other network inference algorithms in both bulk and single-cell synthetic expression data. Lastly, we introduce two case studies, in which we use BTR to improve published Boolean models in order to generate potentially new biological insights.

**Conclusions:**

BTR provides a novel way to refine or reconstruct Boolean models using single-cell expression data. Boolean model is particularly useful for network reconstruction using single-cell data because it is more robust to the effect of drop-outs. In addition, BTR does not assume any relationship in the expression states among cells, it is useful for reconstructing a gene regulatory network with as few assumptions as possible. Given the simplicity of Boolean models and the rapid adoption of single-cell genomics by biologists, BTR has the potential to make an impact across many fields of biomedical research.

**Electronic supplementary material:**

The online version of this article (doi:10.1186/s12859-016-1235-y) contains supplementary material, which is available to authorized users.

## Background

The control of gene expression is tightly regulated by complex gene regulatory networks to achieve cell type specific expression, for example in embryonic [1] and blood development [2]. Moreover, dysregulation of gene expression can lead to disease development, including malignant disease such as leukaemia [3]. A better understanding of gene regulatory networks will therefore not only advance our understanding of fundamental biological processes such as tissue development, but also provide mechanistic insights into disease processes. The earlier versions of high-throughput expression profiling techniques were limited to measuring average gene expression across large pools of cells. By contrast, recent technological improvements have made it possible to perform expression profiling in single cells (See [4] for review). Protocols for the single-cell equivalent of microarray [5], qPCR [6] and RNA sequencing [7] have been developed. One of the key advantages of single cell expression profiling is that it enables the analysis of cells that are rare in number, such as tissue stem cells. In addition, obtaining the expression profiles of single cells is very useful for dissecting the heterogeneity within seemingly homogenous cell populations [2, 8–12].

Because single cell analysis commonly reports expression states for hundreds of individual cells, this unique information offers new opportunities for the development of algorithms that can reconstruct gene regulatory networks. Many network inference algorithms are available [13], which are based on regression, correlation, mutual information and Bayesian networks. However, most of these network inference algorithms only generate a network with static representation of gene interactions. In contrast, changes in network dynamics can be described by using dynamic models, which possess different levels of granularity and precision ranging from the simpler Boolean models to more complex differential equation-based models. More complex models such as differential equation-based models offer high precision predictions, and have been used to describe gene regulatory networks [14–17]. However, such models rely on a higher number of parameters which are often difficult to obtain and verify. In contrast, a Boolean model is one of the simplest models that can describe the dynamics of a system without the need of many parameters (For reviews, see [18, 19]). In a Boolean model, each gene can take a value of 0 or 1, which represents the absence or presence of gene expression respectively. The interactions among genes in a Boolean model are described by Boolean operators like AND, OR and NOT, which closely resembles how biologists describe such interactions. Boolean models were first used to study gene regulatory networks by Kauffman in the 1970s, and since then have been used extensively to study different biological systems [20–23].

While single-cell expression data offers the advantage of capturing expression profiles at single cell resolution, single-cell expression data are noisier than conventional bulk analysis. The technical noise in single-cell expression data arises due to the low amount of input mRNAs in a single cell. This leads to two major sources of technical noise, which are PCR amplification bias and drop-outs [24]. Drop-outs in particular, which represent false negatives where genes are recorded as not expressed due to the low efficiency of mRNA capture from single cells, represent a substantial portion of the technical noise in single-cell expression data. Therefore, network inference techniques that are robust to the effect of drop-outs are required when reconstructing networks using single-cell expression data. Boolean models are relatively robust to the presence of drop-outs due to the binarisation of expression values. Two recent studies reported algorithms for inferring Boolean models from single-cell expression data [2, 25]. Chen et. al. developed SingCellNet, which uses a genetic algorithm to construct probabilistic Boolean models from expected trajectories through cell states [25]. However, SingCellNet is restricted to small networks with less than 10 genes, and it only determines the network structure and transition probabilities from single-cell expression data. The Boolean rules in SingCellNet are constructed via manual curation from the literature. In another study, SCNS was developed by Moignard et. al. to infer an asynchronous Boolean model by analysing trajectories through a state transition graph [2]. In order to infer a Boolean model using SCNS, a connected state transition graph is required, which can be difficult to obtain from single-cell expression data. This is because the higher the number of genes to be included in SCNS, the more cells will be required to build a connected state transition graph. In addition, SCNS can only infer network structure by using discretised expression data, which not only leads to the loss of information, but also makes SCNS sensitive to the discretisation method used. Lastly, both SingCellNet and SCNS rely on known general trajectories through the cell states, which require single-cell expression data from at least two cell types with known relationships.

Here, we present a model learning algorithm BTR (BoolTraineR), that is able to reconstruct and train asynchronous Boolean models using single-cell expression data. BTR differs from other algorithms described above in that it can infer both network structure and Boolean rules without needing information on trajectories through cell states. We developed a scoring function based on the Boolean framework, which performed favourably in comparison to a scoring function for Bayesian network. We show that BTR outperforms other network inference algorithms when initial networks are supplied. Lastly, we demonstrate the capability of BTR by training Boolean models using single-cell qPCR and RNA-Seq data from haematopoietic studies.

## Results and discussion

### A framework for scoring Boolean models with single cell expression data

**Fig. 1:**
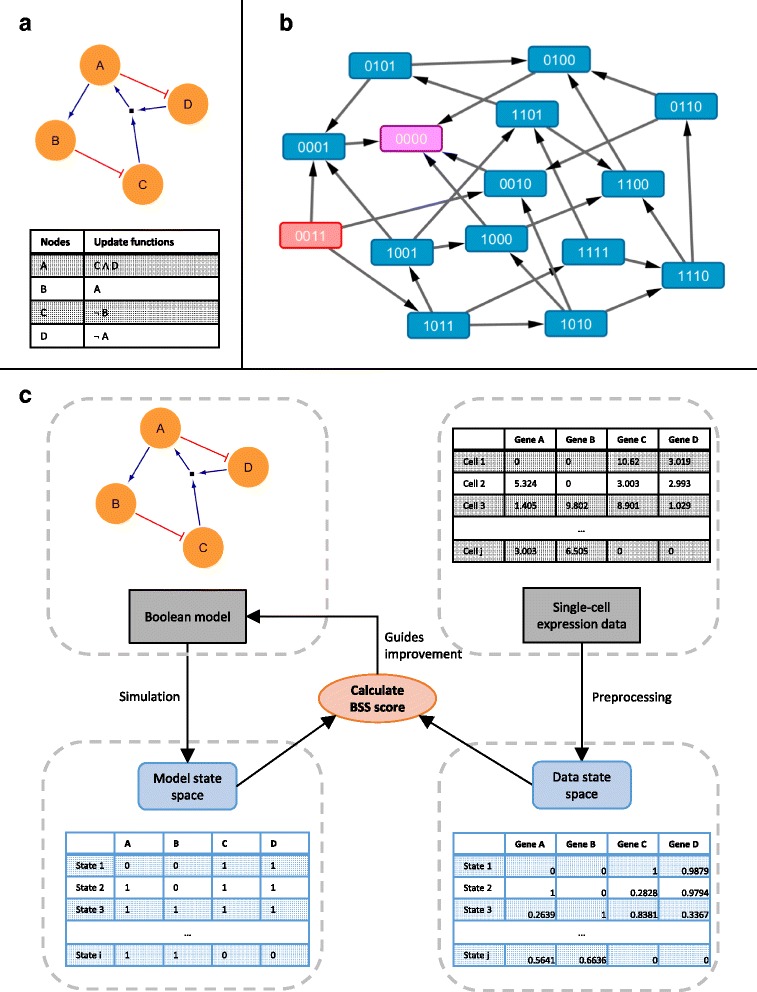
Boolean model, asynchronous simulation and the framework underlying BTR. **a** A Boolean model can be expressed graphically in terms of nodes and edges, as well as in tabular form in terms of update functions. Note that the small black node refers to AND interaction. **b** The asynchronous update scheme is best explained with the use of a graph representation of state space, in which each connected state differs in only one node. Starting from the initial state *s*_1_ = {0, 0, 1, 1} and evaluated using the update functions in (**a**), asynchronous simulation produces a model state space with 15 states. The initial state is shown in *red node*, while the final steady state is shown in *pink node*. **c** The framework underlying BTR. A Boolean model can be simulated to give a model state space, while a single-cell expression data can be preprocessed to give a data state space. Boolean state space scoring function can then calculate the distance score between the model and data state spaces. Lastly, BTR uses the computed distance score to guide the improvement of the Boolean model through an optimisation process that minimises the distance between model and data state spaces

A Boolean model *B* is made up of *n* genes *x*_1_, …, *x*_*n*_ and *n* update functions *f*_1_, …, *f*_*n*_ : {0, 1}^*n*^ → {0, 1} each associated with a gene (Fig. 1a). Each gene can take a value *x* ∈ {0, 1}, which represents the absence or presence of gene expressions. Each update function *f* is expressed in terms of Boolean logic by specifying the relationships among genes *x*_1_, …, *x*_*n*_ using Boolean operators AND (∧), OR (∨) and NOT (¬). The main difference of asynchronous with other Boolean models is the update scheme used during simulation. An asynchronous Boolean model uses the asynchronous update scheme, which specifies that at most one gene is updated between two consecutive states. Asynchronous updating is critical when modelling developmental systems that generate distinct differentiated cell types from a common progenitor, because synchronous updating generates fully deterministic models and therefore cannot capture the ability of a stem cell to mature into multiple different tissue cells.

A state in a Boolean model *B* is represented by a Boolean vector *s*_*t*_ = {*x*_1*t*_, …, *x*_*nt*_} at simulation step *t*. States can be generated from an initial state by systematically changing one variable at each step according to the Boolean function associated with that variable. If a state has already been encountered earlier, it is ignored. This results in a directed graph of states as exemplified in Fig. 1b, where any two connected states change in just one variable. When all the states in the directed graph are taken together, they represent a model state space. The initial state used in a simulation can be obtained from the expression values at time = 0 for a time-series expression dataset, or it can be obtained from the expression values of known parental cell types.

Of note, the model state space of an asynchronous Boolean model closely resembles a single-cell expression data. The model state space contains predicted expression states that are dictated by a known gene network that underlies a Boolean model; while the single-cell expression data can be viewed as a data state space which contains observed expression states that are dictated by an unknown gene network. By fine-tuning the network rules underlying the Boolean model, it should be possible to produce a predicted model state space that closely resembles an observed data state space, thereby allowing us to reconstruct the unknown gene network. BTR uses this framework to reconstruct a Boolean model from single-cell expression data (Fig. 1c). In this framework, a Boolean model is represented by its model state space, while a single-cell expression dataset is represented by its data state space. By utilising the novel Boolean state space (BSS) scoring function (See Methods), BTR evaluates how well a particular Boolean model explains the single-cell expression data by scoring the model state space with respect to the data state space. During the model training process, BTR uses a swarming hill climbing strategy to generate minimally modified Boolean models based on an initial Boolean model. These minimally modified Boolean models are then scored using the BSS scoring function, and BTR selects the best scoring Boolean models for the next iteration. By performing this process iteratively, BTR reconstructs the asynchronous Boolean model that can best explain a single-cell expression dataset.

### Boolean state space scoring represents a powerful scoring function for Boolean models

How well BTR performs depends heavily on the performance of the BSS scoring function. Among different modelling frameworks, the Bayesian network framework is known to possess several well-established scoring functions that evaluate how well a particular network fits a given dataset. These scoring functions include log-likelihood, Bayesian information criterion (BIC), Bayesian Dirichlet and K2 (See [26, 27] for reviews). Since expression data have continuous values for gene expressions, we have selected the BIC scoring function, which can handle continuous variables, as a scoring function from the Bayesian network framework for comparison purpose.

BSS and BIC scoring functions were evaluated using synthetic data. The true network and expression data in the synthetic data were generated using GeneNetWeaver [28], which is also used in the DREAM5 network inference challenge [13]. In order to simulate the zero-inflated property of single-cell expression data due to the presence of drop-outs, we introduced zero inflation into the synthetic data as described in the Methods section. An ideal scoring function should give an increasing distance score, as the evaluated network becomes increasingly different from the true network. In order to test this, we generated a list of modified networks that are increasingly different from the true network in terms of edges. As Bayesian networks and Boolean frameworks imposed different network structure constraints, the modified networks were generated separately to give a list of modified Bayesian networks and another list of modified Boolean networks. Although the modified Bayesian and Boolean networks are not identical, they possess the same number of differing edges when compared to the true network, ranging from 2 edges up to 40 differing edges. Five independent benchmark data, each with a different true network, true data and modified models, were used in the evaluation of scoring functions.

**Fig. 2:**
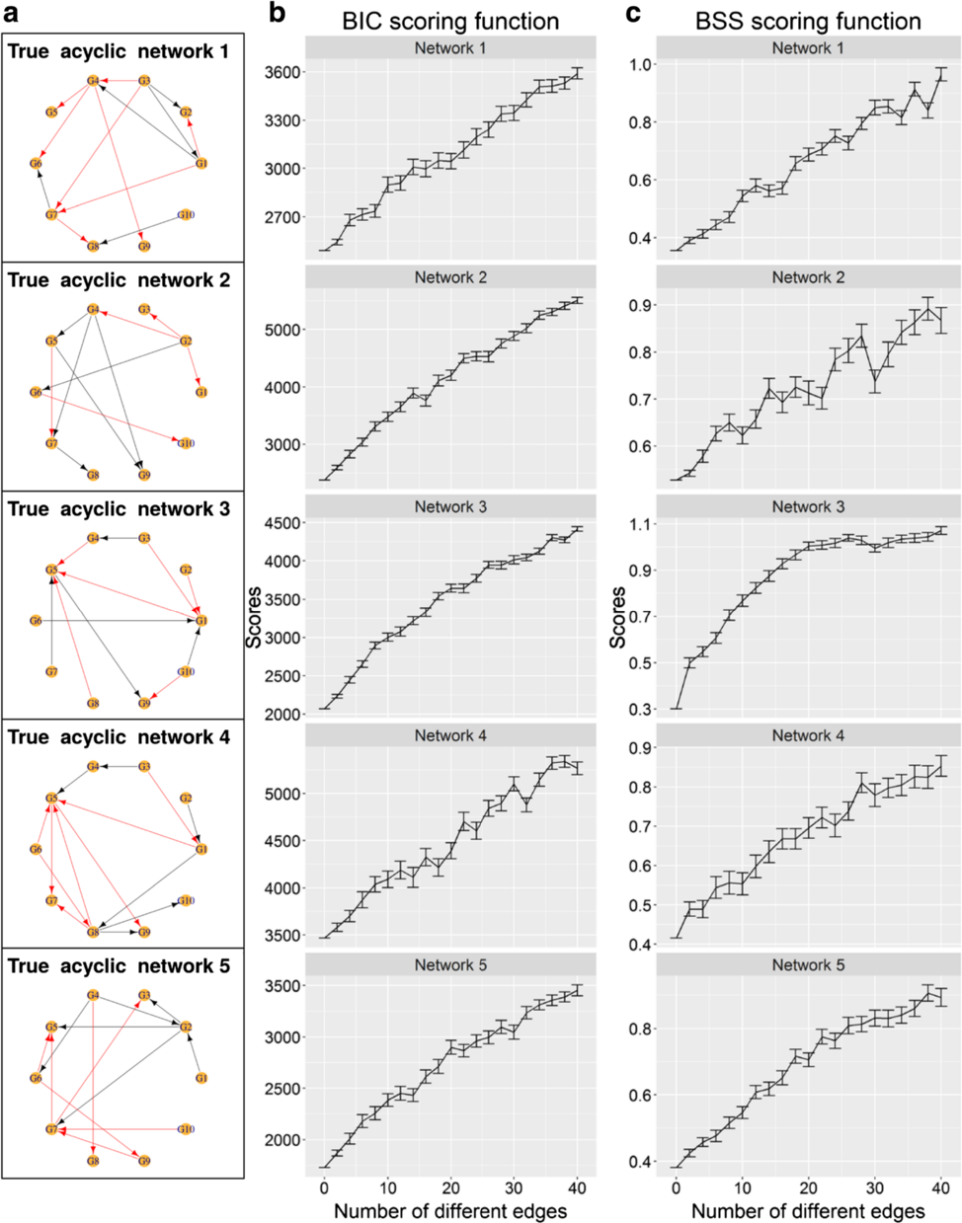
BSS scoring function compares favourably with BIC scoring function on acyclic networks. **a** Acyclic networks generated from GeneNetWeaver that are designated as the true acyclic networks. Each node corresponds to a gene. *Black edges* indicate activation interactions, while red edges indicate inhibition interactions. Mean distance scores computed using **b** BIC scoring function and **c** BSS scoring function for modified networks that are increasingly different from the true network in terms of edges using zero-inflated synthetic expression data. The modified networks contain from two edges up to forty different edges when compared with the true network. Each data point is the mean distance score of 100 different random modified networks that contain the same number of different edges with respect to the true network. The error bar is the standard error of the mean

By evaluating networks using zero-inflated synthetic data, both BSS and BIC scoring functions performed well when acyclic networks are considered (Fig. 2). Both scoring functions were able to give increasing distance scores as the underlying networks become increasingly different from the true network. The BSS scoring function achieves this by considering the input expression data as a data state space, and then computing the distance score by comparing the data state space with the model state space simulated from a given network. It is expected that as a network become increasingly different, its model state space will become increasingly different from the data state space, which is reflected in the distance score as shown in Fig. 2c. To the best of our knowledge, this is the first time a scoring function that is based entirely on the Boolean modelling framework has been demonstrated to give comparable performance with a scoring function for Bayesian networks.

**Fig. 3:**
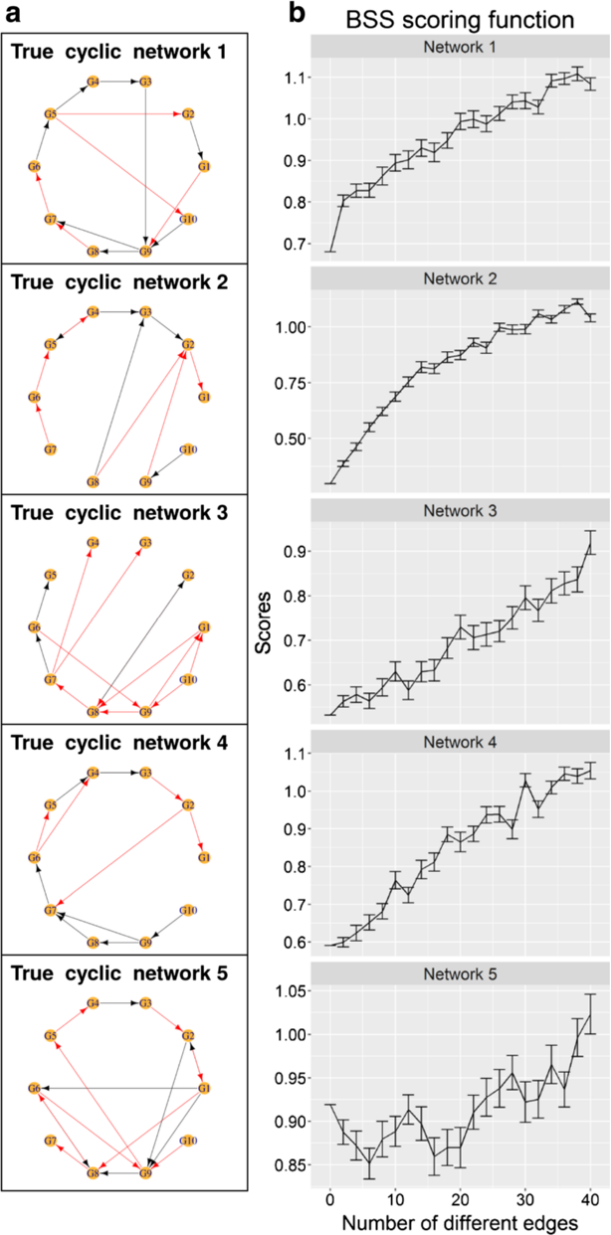
BSS scoring function is able to calculate distance scores for cyclic networks. **a** Cyclic networks generated from GeneNetWeaver that are designated as the true cyclic networks. Each node corresponds to a gene. *Black edges* indicate activation interactions, while *red edges* indicate inhibition interactions. **b** Mean distance scores computed using BSS scoring function for modified networks that are increasingly different from the true network in terms of edges using zero-inflated synthetic expression data. The modified networks contain from two edges up to forty different edges when compared with the true network. Each data point is the mean distance score of 100 different random modified networks that contain the same number of different edges with respect to the true network. The error bar is the standard error of the mean

As indicated in the results for Network 2 (Fig. 2c), the BSS scoring function is dependent on the underlying true network structure in certain cases and will work better on distinguishing networks that are very different. However the BSS scoring function has a distinct advantage over scoring functions for Bayesian networks. The Bayesian networks are known to impose relatively strict constraints on permissible network structures, in particular Bayesian networks are not allowed to contain any cyclic network structure. Therefore scoring functions for Bayesian networks cannot be used to evaluate cyclic networks. Cyclic networks are ubiquitous in biological systems, in which cyclic motifs can be present in the form of negative and positive feedback loops. Boolean models on the other hand are allowed to have any number of cyclic motifs in the networks. Therefore, the BSS scoring function can be used to compute scores for cyclic networks. By using another five independent benchmark data with true networks that contain at least one cycle, the distance scores for modified networks were computed (Fig. 3). The distance scores for cyclic networks have more fluctuations compared to acyclic networks due to the presence of cyclic motifs. However, the general trend where the distance scores increase as the underlying networks become increasingly different from the true network was still observed.

**Fig. 4:**
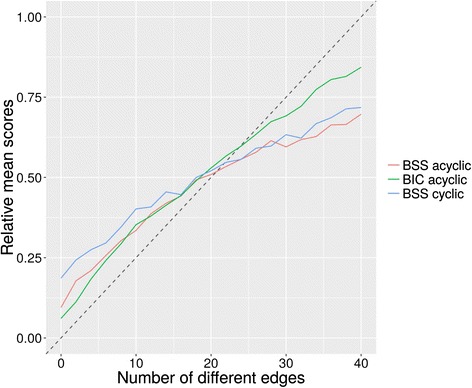
Summary of BIC and BSS scoring functions. Mean scores have been calculated across all networks (five acyclic networks and five cyclic networks) for BIC and BSS scoring functions calculated using zero-inflated synthetic expression data. All scores have been standardised for comparison purpose, such that the scores range from 0 to 1

We have also evaluated the series of acyclic and cyclic networks using non zero-inflated data (Additional file 1: Figure S1 & Additional file 2: Figure S2). When the results computed with non zero-inflated data are compared to the results computed using zero-inflated data, we can see that zero-inflation has no effect on BIC scores and a small effect on BSS scores that does not affect the general trend (Additional file 3: Figure S3). In summary, the relative mean scores that average across the results of all networks (Fig. 4) show that although the BIC scoring function performs slightly better than the BSS scoring function, the BSS scoring function has the advantage that it can evaluate cyclic networks.

### BTR accurately infers the networks underlying synthetic datasets

Next, we compared the network inference performance of BTR with other well-known network inference algorithms. Two search algorithms guided by the BSS Boolean and BIC Bayesian network scoring functions were included in the comparison, indicated as BTR and BIC respectively. The search algorithms used for both scoring functions are both based on hill climbing. The additional network inference algorithms included in the comparison are BestFit [29], ARACNE [30], CLR [31], bc3net [32], GeneNet [33] and Genie3 [34] (See Methods for brief details on the algorithms).

**Fig. 5:**
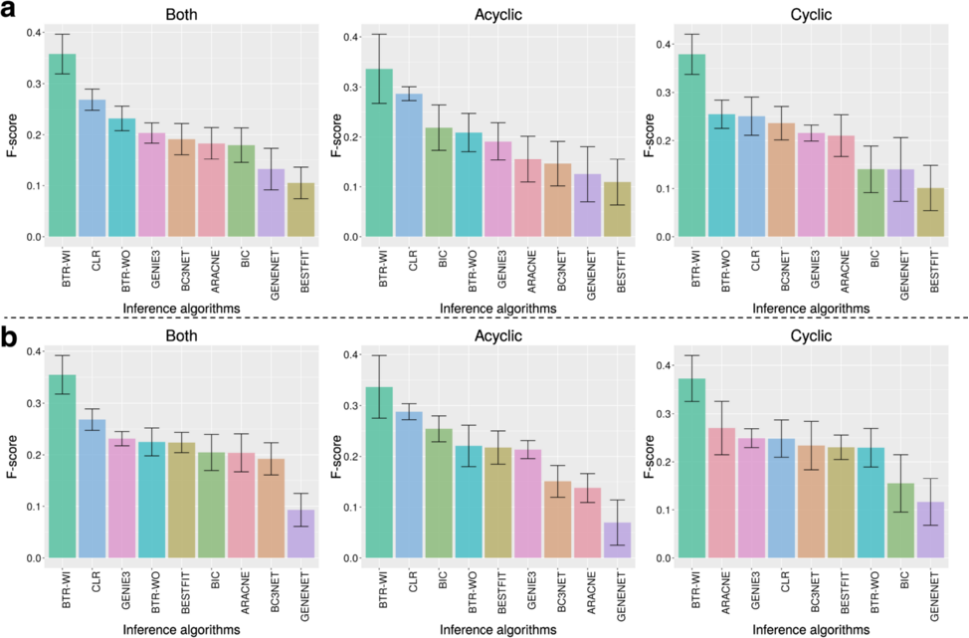
BTR outperforms other network inference algorithms. Mean F-scores of network inference algorithms inferred using **a** non zero-inflated synthetic data and **b** zero-inflated synthetic data. Ten true synthetic networks (Five each for acyclic and cyclic networks) were used in the assessment of these network inference algorithms. Plots titled ‘Both’ show the combined results of acyclic and cyclic network inference. The error bar is the standard error of the mean

By using the same synthetic networks, as well as both non zero-inflated and zero-inflated synthetic data, we performed network inference using the synthetic expression data alone without any extra information. In contrast to the DREAM5 challenge [13] which also provides perturbed expression data, only a single type of expression data is provided to all the network inference algorithms, which is the wild type time course expression data in steady state. For BTR, besides performing inference with only expression data (indicated as BTR-WO), we also performed inference with both expression data and initial networks (indicated as BTR-WI) to show that BTR is able to use initial networks with known network structure to improve the inference process. The initial networks are generated randomly to contain 18 edges that are different compared with the true networks. The performance of the network inference algorithms is assessed in terms of F-scores [35] (Fig. 5). In order to allow comparisons on the performance across all network inference algorithms tested, we calculated the F-scores based only on the presence or absence of edges, while ignoring any additional information such as the types of edges.

In terms of acyclic networks, the results show that the top inference algorithms using either non zero-inflated or zero-inflated data are BTR-WI, CLR, BIC and BTR-WO. As for cyclic networks, the top inference algorithms differ between using non zero-inflated and zero-inflated data. BTR-WI, BTR-WO, CLR and BC3NET gave the best performance with non zero-inflated data, while BTR-WI, ARACNE, GENIE3 and CLR gave the best performance with zero-inflated data. When all results are taken together, BTR-WI, CLR, BTR-WO and GENIE3 gave the best performance overall. Note that the ranking of network inference algorithms in this study differs from the ranking of the DREAM study because different scoring criteria are used (F-score is used here as opposed to the area under the precision-recall (AUPR) and receiver operating characteristic (AUROC) curves in the DREAM study); and the DREAM study was done using multiple types of synthetic data, such as expression data with gene perturbations. In general, the presence of drop-outs affects the performance of network inference algorithms in different ways (Fig. 5b). In cases such as bc3net and GeneNet, their performance decreases when drop-outs are present, while the impact of drop-outs on the performance of BTR is minimal. Interestingly, the performance of BestFit increases with the presence of drop-outs, possibly due to better binarisation of data due to the information given by drop-outs. As both BTR and BestFit are algorithms for inferring Boolean model, this result provides further support that Boolean models are robust to the presence of drop-outs in single-cell expression data.

When given an initial network as in BTR-WI, the BTR algorithm was able to perform very well in locating the true network. While the performance of the BTR algorithm without an initial network (BTR-WO) is comparable with other inference algorithms, BTR-WO scored less well compared to BTR-WI. This indicates that the greedy hill climbing search strategy implemented in BTR may not be able to traverse the solution space efficiently without any initial information. Taken together, while BTR can be used for reconstructing network models without initial information, BTR performed the best when it is used to train and improve on existing networks that contain a partially true structure. It is also worth noting that BTR produced a dynamic model with a directed underlying static network, in contrast to most other algorithms such as CLR that only produce an undirected static network.

### BTR predicts gene interactions by training haematopoietic Boolean models

**Fig. 6:**
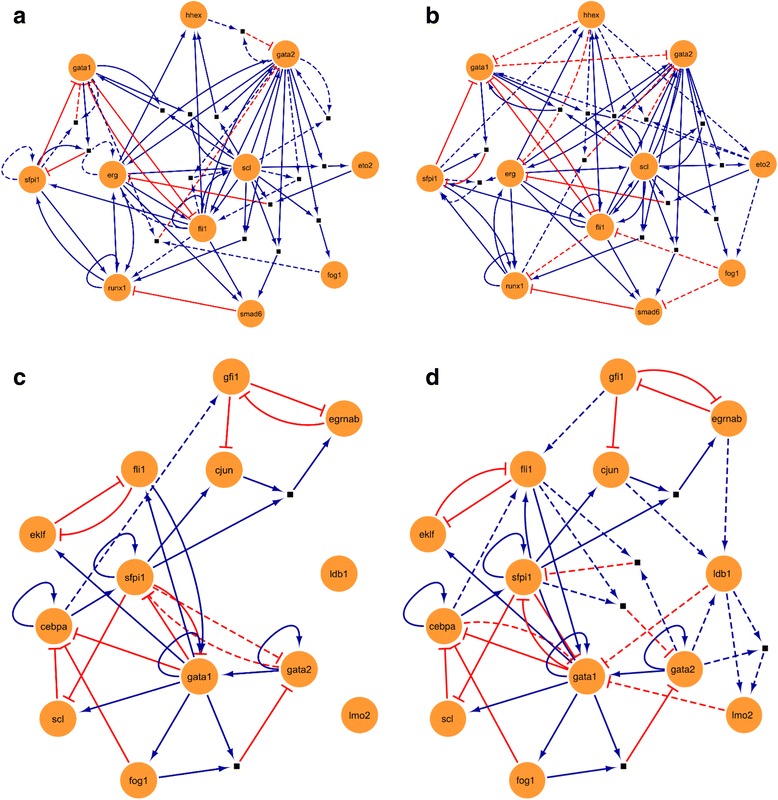
BTR predicts gene interactions by training the Bonzanni and Krumsiek Boolean models. **a** Original Bonzanni model. **b** Trained Bonzanni model. **c** Original Krumsiek model. **d** Trained Krumsiek model. *Round orange nodes* indicate genes, *square black nodes* indicate AND gates that combine the two input gene interactions. *Blue edges* indicate activation interactions, *red edges* indicate inhibition interactions. *Dashed lines* in the original models indicate edges that are present in the original models, but are removed in the trained models. *Dashed lines* in the trained models indicate edges that are added to the trained models and are not present in the original models

We next wanted to apply BTR to biological data to evaluate its utility to biologists. Haematopoiesis research has provided many paradigms for modern biological research, and was one of the first fields to embrace single cell expression profiling [5, 36, 37]. Moreover, literature curated Boolean network models have been reported both for blood stem cell maintenance and blood progenitor differentiation [38, 39]. The single-cell expression data used here includes single-cell qPCR and single-cell RNA-Seq data, which are both obtained from [10]. The two Boolean models will be referred to as the Bonzanni model [39] (Fig. 6a) and the Krumsiek model [38] (Fig. 6c). Both models had been constructed via manual literature curation by the authors of the original papers. The Bonzanni model aimed to capture haematopoietic stem cell (HSC) self-renewal capacity, while the Krumsiek model describes the differentiation process of the erythro-myeloid lineage in haematopoiesis.

**Fig. 7:**
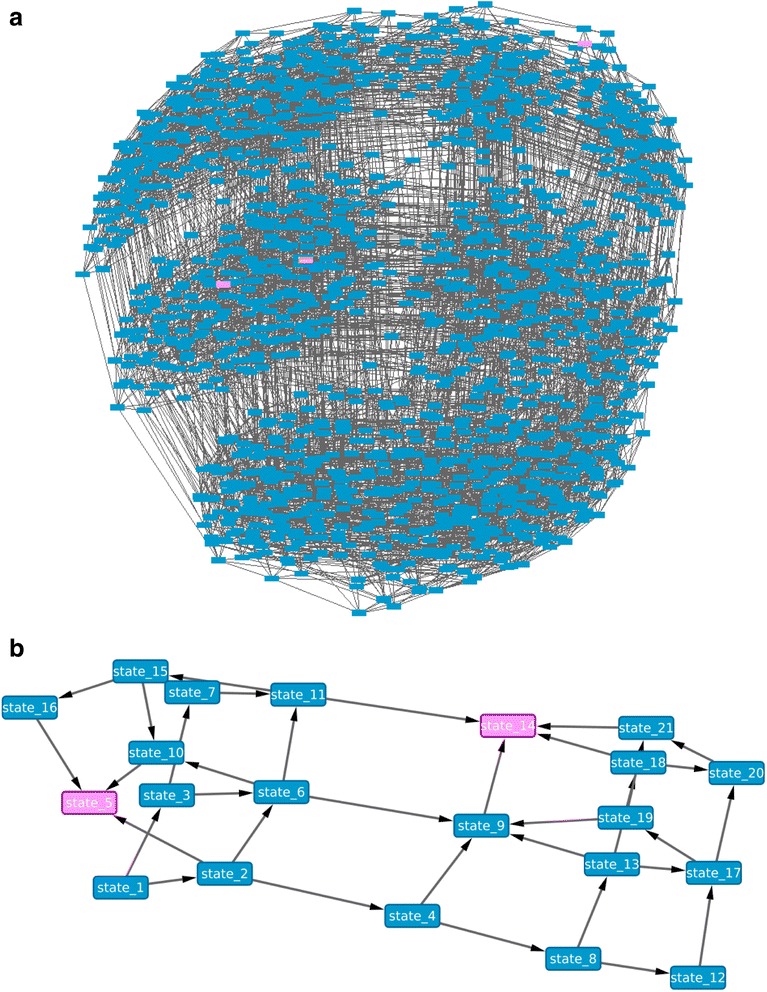
State spaces for the trained Bonzanni and Krumsiek Boolean models. **a** State space of trained Bonzanni model. **b** State space of trained Krumsiek model. *Blue nodes* represent transitional model states, while *pink nodes* represent steady model states. Each *arrow* indicates transitions among states

**Fig. 8:**
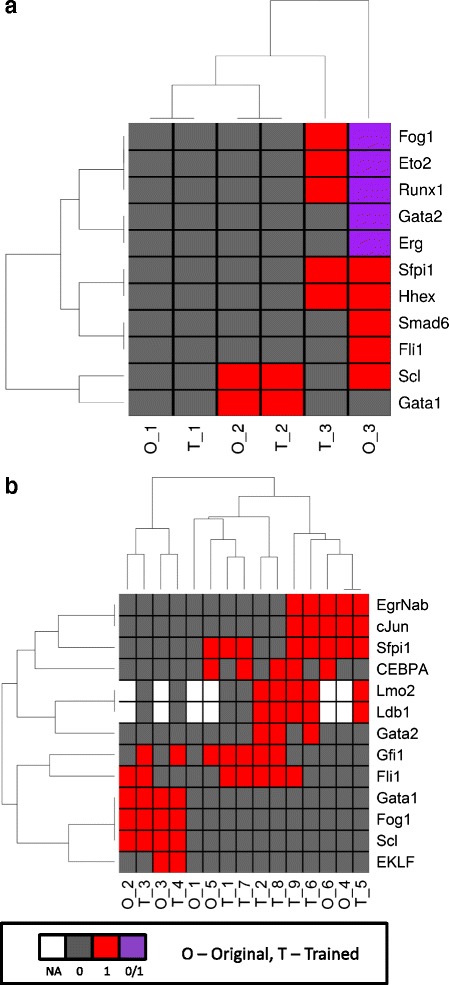
Steady states for the Bonzanni and Krumsiek Boolean models. **a** Steady states of Bonzanni models. Both original and trained models contain two point steady states and one cyclic steady state each. **b** Steady states of Krumsiek models. Original model contains six point steady states, while trained model contains nine point steady states. *Black box* indicates expression is absent (i.e. 0), *red box* indicates expression is present (i.e. 1), *purple box* indicates expression can be absent or present (i.e. 0 or 1). *White box* is used to indicate that the additional genes included in trained Krumsiek model that are not present in the original Krumsiek model

We firstly trained the Bonzanni model using single-cell RNA-Seq data collected from HSCs. Compared to the original model, the resulting trained Bonzanni model (Fig. 6b) shows the deletions of ten gene interactions and the additions of thirteen gene interactions (Table 1). The state space of the trained Bonzanni model contains 1486 states when simulated using the initial state used in the original study (Fig. 7a). Of note, there are many densely connected transitional states in the state space, which may be related to the complexity of cell fate decision making processes in multipotent progenitor cells. Steady state analysis performed showed that the steady states of the trained Bonzanni model are almost identical to the steady states of the original Bonzanni model (Fig. 8a), except with the absence of cyclic steady states. The authors suggested that the cyclic steady states in the original Bonzanni model correspond to the self-renewal maintenance loop in HSCs, which is not present in our trained model possibly because the number of cells profiled by single-cell RNA-seq is not enough to sufficiently capture the HSC self-renewal expression signature. We then trained the Krumsiek model by using single-cell qPCR data collected from over 450 cells along the erythro-myeloid lineage, which includes common myeloid progenitors, granulocyte-monocyte progenitors and myeloid-erythroid progenitors. In order to demonstrate that BTR can be used in cases where we may want to extend a current Boolean model by adding more genes to it, we have used BTR to train and add two additional genes to the Krumsiek model. The resulting trained Krumsiek model (Fig. 6d) contains three deleted gene interaction and twelve added gene interactions (Table 1) when compared to the original Krumsiek model. For the two additional genes *Ldb1* and *Lmo2*, BTR has predicted gene interactions among *Ldb1*, *Lmo2*, *Fli1*, *Gata1* and *Gata2*. Previous studies have shown that genome-wide binding profiles for *Lmo2*, *Gata2* and *Fli1* show significant overlaps [40], and that *Ldb1* also occupies nearly all of the binding sites of *Gata2* [41], consistent with a model where these TFs engage in combinatorial interactions. The state space of the trained Krumsiek model contains 21 states when simulated using the initial state used in the original study (Fig. 7b). The two steady states reachable in this state space may correspond well to cell populations that are primed for the erythrocyte and myeloid lineage divergence. When examining the steady states reachable from all possible initial states, the trained Krumsiek model produces additional steady states when compared with the original model due to the addition of two extra genes (Fig. 8b), which may correspond to intermediate cell types along the erythro-myeloid differentiation pathway.

**Table 1:** Differences in gene interactions between original and trained Bonzanni and Krumsiek models

Models	Gene interactions	Suggested modifications to original model
Bonzanni	*Gata2* and *Hhex* inhibits *Gata2*	Deletion
	*Scl* and *Gata2* activates *Scl*	Deletion
	*Fli1* and *Gata2* activates *Scl*	Deletion
	*Gata2* and *Scl* activates *Fli1*	Deletion
	*Fli1* activates *Runx1*	Deletion
	*Fli1* activates *Erg*	Deletion
	*Erg* activates *Erg*	Deletion
	*Gata1* and *Fog1* inhibits *Gata2*	Deletion
	*Sfpi1* and *Gata1* inhibits *Gata1*	Deletion
	*Sfpi1* activates *Sfpi1*	Deletion
	*Sfpi1* and *Erg* activates *Sfpi1*	Addition
	*Hhex* and *Runx1* inhibits *Gata2*	Addition
	*Eto2* and *Hhex* inhibits *Gata2*	Addition
	*Sfpi1* activates *Hhex*	Addition
	*Gata1* inhibits *Gata2*	Addition
	*Fog1* inhibits *Smad6*	Addition
	*Fog1* inhibits *Fli1*	Addition
	*Eto2* activates *Gata1*	Addition
	*Eto2* activates *Fog1*	Addition
	*Hhex* inhibits *Gata1*	Addition
	*Hhex* activates *Eto2*	Addition
	*Hhex* inhibits *Erg*	Addition
	*Fli1* inhibits *Runx1*	Addition
Krumsiek	*Cebpa* activates *Gfi1*	Deletion
	*Gata2* inhibits *Sfpi1*	Deletion
	*Sfpi1* inhibits *Gata2*	Deletion
	*Gfi1* activates *Fli1*	Activation
	*Cebpa* activates *Fli1*	Activation
	*Fli1* and *Gata2* inhibits *Sfpi1*	Activation
	*Fli1* and *Sfpi1* inhibits *Gata2*	Activation
	*Cebpa* inhibits *Gata1*	Activation
	*EgrNab* activates *Ldb1*	Activation
	*cJun* activates *Ldb1*	Activation
	*Ldb1* inhibits *Gata1*	Activation
	*Gata2* activates *Ldb1*	Activation
	*Ldb1* activates *Lmo2*	Activation
	*Ldb1* and *Gata2* activates *Lmo2*	Activation
	*Lmo2* inhibits *Gata1*	Activation

Other gene interactions that were not modified are not listed in this table. Each gene interaction corresponds to an edge on the network

Taken together, the result suggests that both the trained Bonzanni and Krumsiek models have been trained by BTR to predict new gene interactions which give rise to interesting state spaces and steady state properties. Note that the state space of the trained Bonzanni model is substantially larger than the state space of the trained Krumsiek model due to the denser interactions among genes and a lower proportion of inhibitory edges in the trained Bonzanni model (Additional file 4: Figure S5).

## Conclusions

We have developed the BTR model learning algorithm for training asynchronous Boolean models using single-cell expression data. The key component in BTR is a novel Boolean state space (BSS) scoring function, which BTR uses to infer a Boolean model through an optimisation process. We have shown that the new BSS scoring function is capable of giving meaningful scores to networks when compared with the BIC scoring function for Bayesian networks. We then showed that when compared to other network reconstruction algorithms, BTR gave the best result when initial networks were provided. In two case studies, we have demonstrated that BTR is capable of suggesting modifications to existing Boolean models based on information from single-cell qPCR and RNA-Seq data. Finally, we anticipate BTR to be a useful addition to the current toolbox for processing and understanding single-cell expression data, as it provides significant new capabilities for regulatory network modelling in a user-friendly way.

## Methods

### Definitions

A Boolean model *B* consists of *n* genes *x*_1_, …, *x*_*n*_ and *n* update functions *f*_1_, …, *f*_*n*_ : {0, 1}^*n*^ → {0, 1}, with each *f*_*i*_ being associated with gene *x*_*i*_ (Fig. 1a). Each gene *x*_*i*_ corresponds to a binary variable representing the expression value of the gene, i.e. *x* ∈ {0, 1}. Gene *x*_*i*_ is a target gene when it acts as a response variable and an input gene when it acts as a predictor variable. Each update function *f*_*i*_ can be evaluated to give a value to a target gene *x*_*i*_, and is expressed in terms of Boolean logic by specifying the relationships among a subset of the input genes *x*_1_, …, *x*_*n*_ using Boolean operators AND (∧), OR (∨) and NOT (¬). An update function *f*_*i*_ consists of an activation clause and an inhibition clause in the form of:

$$ \left(\mathit{\mathsf{activation}}\kern0.5em \mathit{\mathsf{clause}}\right) \wedge \neg \kern0.5em \left(\mathit{\mathsf{inhibition}}\kern0.5em \mathit{\mathsf{clause}}\right) $$

Each clause is individually expressed in disjunctive normal form, (*u*_1_) ∨ (*u*_2_) ∨ (*u*_3_) ∨ … ∨ (*u*_*n*_), where *u* represents a slot which can either take in a single input gene *x*_*i*_ or a conjunction of two input genes *x*_*i*_ ∧ *x*_*i* + 1_. An example update function *f*_1_(*s*_*t*_) for a target gene *x*_1_ with an input state *s*_*t*_ is given below:

$$ {\mathit{\mathsf{x}}}_{\mathsf{1}} = {\mathit{\mathsf{f}}}_{\mathsf{1}}\left({\mathit{\mathsf{s}}}_{\mathit{\mathsf{t}}}\right)=\left(\left({\mathit{\mathsf{x}}}_{\mathsf{3}}\wedge {\mathit{\mathsf{x}}}_{\mathsf{4}}\right)\right)\wedge \neg \kern0.5em \left(\left({\mathit{\mathsf{x}}}_{\mathsf{5}}\right)\vee \left({\mathit{\mathsf{x}}}_{\mathsf{2}}\wedge {\mathit{\mathsf{x}}}_{\mathsf{9}}\right)\right) $$

A few constraints are imposed on the update functions during model learning in BTR. Firstly, the update function allows a conjunction of up to two input genes in each slot *u*. Secondly, each input gene *x*_*i*_ can only be present in a single update function once, but the same input gene *x*_*i*_ can be present in multiple update functions. Thirdly, a user is able to specify a soft limit on the number of input genes (i.e. in-degree) allowed per update function, where the default in BTR is 6 in-degree per gene. Lastly, by default no self-loop is allowed in BTR.

A model state given by a Boolean model *B* is represented by a Boolean vector *s*_*t*_ = {*x*_1*t*_, …, *x*_*nt*_} at simulation step *t*. A model state space *S* represents the set of all model states *s*_*t*_ reachable from an initial model state *s*_1_, i.e. *S* = {*s*_1_, …, *s*_*t*_}. *S* can be obtained by simulating the model *B* starting from an initial model state *s*_1_ using the asynchronous update scheme. The asynchronous update scheme specifies that at most one gene is updated between two consecutive states (Fig. 1b). Assuming we have a model state *s*_t_ which is not a steady state, there will be *i* (*i* ≥ 1) genes in *s*_*t*_ such that *x*_*it*_ ≠ *f*_*i*_(*s*_*t*_). Therefore at simulation step *t* + 1, *s*_*t* + 1_ would have *i* possible configurations *s*_*t* + 1_^*i*^, where *s*_*t* + 1_^*i*^ = {*x*_1*t*_, …, *f*_*i*_(*s*_*t*_), …, *x*_*nt*_}. This simulation is repeated until it reaches a steady state. By definition, steady states are a set of states whose destination states also belong to the same set. That is, a steady state may be a single model state *s*_*t*_, or it may consist of a cyclic sequence of model states *s*_*t*_, …, *s*_*t* + *j*_.

The single-cell expression data used in this study are each a matrix consisting of *n* individual genes in the columns and *k* individual cells in the rows. The expression data are normalised and standardised to give *y*_*kn*_ ∈ [0, 1]. A data state *v*_*k*_ = {*y*_1_, …, *y*_*n*_} represents the expression state of cell *k* for *n* genes that are observed in the cell. A data state space *V* = {*v*_1_, …, *v*_*k*_} represents the set of all data states that are observed in an experiment.

### BTR model learning

The aim of BTR is to identify a Boolean model *B* with *x*_*n*_ genes and *f*_*n*_ update functions, that can produce a model state space which closely resembles an independent single-cell expression data (i.e. data state space). Note that model state space and data state space are defined in a similar way, the only difference being that the *n* genes take continuous values in [0, 1] within a data state, while the *n* genes take binary values 0 and 1 in a model state. The distance between model and data state spaces is measured by the pairwise distance between pairs of model and data states, as stated in the scoring function (See below). By iteratively modifying an initial Boolean model *B*_1_, the distance between the model and data state spaces can be minimised until a resulting final Boolean model *B*_*f*_ with less distance is obtained.

BTR performs model learning by utilising techniques in discrete optimisation framework. In any optimisation problem, there are two important components, namely a scoring function and a search strategy.

#### BSS Scoring function in BTR

The scoring function used in BTR is a novel scoring function we developed, termed as Boolean state space (BSS) scoring function. BSS scoring function *g*(*S*, *V*) is a distance function, which consists of a base distance variable and two penalty variables. *g*(*S*, *V*) is given by:

$$ \mathit{\mathsf{g}}\left(\mathit{\mathsf{S}},\ \mathit{\mathsf{V}}\right)=\mathit{\mathsf{h}}\left(\mathit{\mathsf{S}},\ \mathit{\mathsf{V}}\right) + {\lambda}_{\mathsf{1}}{\varepsilon}_{\mathsf{1}} + {\lambda}_{\mathsf{2}}{\varepsilon}_{\mathsf{2}} $$

Where *h*(*S*, *V*) = base distance, *ε* = penalty variable, *λ* = constant for penalty variable.

The base distance *h*(*S*, *V*) is given by the following equation. To prevent multiple model states from matching to a single data state, one-to-one matching between model and data states is enforced if the number of data states, *N*_*v*_, are more than or equal to the number of model states, *N*_*s*_, i.e. *N*_*v*_ ≥ *N*_*s*_. For cases where *N*_*v*_ < *N*_*s*_, one-to-one matching between model and data states is enforced greedily up until the point where every data states have been assigned a matching model state, then non-unique matching will occur for the remaining model states with respect to each corresponding data state with the minimum distance.

$$ \mathit{\mathsf{h}}\left(\mathit{\mathsf{S}},\ \mathit{\mathsf{V}}\right) = \frac{{\displaystyle {\sum}_{\mathit{\mathsf{t}}=\mathsf{1}}^{{\mathit{\mathsf{N}}}_{\mathit{\mathsf{s}}}}}\mathit{\mathsf{m}}\mathit{\mathsf{i}}{\mathit{\mathsf{n}}}_{\mathit{\mathsf{k}}=\mathsf{1}}^{\mathit{\mathsf{N}}\mathit{\mathsf{v}}}\left(\mathit{\mathsf{d}}\left({\mathit{\mathsf{s}}}_{\mathit{\mathsf{t}}},\ {\mathit{\mathsf{v}}}_{\mathit{\mathsf{k}}}\right)\right)}{{\mathit{\mathsf{N}}}_{\mathit{\mathsf{s}}}\ \mathit{\mathsf{n}}} $$

Where $\mathit{\mathsf{d}}\left({\mathit{\mathsf{s}}}_{\mathit{\mathsf{t}}},\ {\mathit{\mathsf{v}}}_{\mathit{\mathsf{k}}}\right)$ = pairwise distance between each model state *s*_*t*_ and data state *v*_*k*_ (0 ≤ *d*(*s*_*t*_, *v*_*k*_) ≤ 1), *N*_*s*_ = number of model states, *N*_*v*_ = number of data states, *n* = number of genes.

The distance between model state *s*_*t*_ and data state *v*_*k*_, *d*(*s*_*t*_, *v*_*k*_), is defined as the sum of the absolute differences between values of each gene *i* in model state *s*_*t*_ and data state *v*_*k*_.

$$ \mathit{\mathsf{d}}\left({\mathit{\mathsf{s}}}_{\mathit{\mathsf{t}}},\ {\mathit{\mathsf{v}}}_{\mathit{\mathsf{k}}}\right) = {\displaystyle \sum_{\mathit{\mathsf{i}}=\mathsf{1}}^{\mathit{\mathsf{n}}}}\left|\ {\mathit{\mathsf{x}}}_{\mathit{\mathsf{t}}\mathit{\mathsf{i}}} - {\mathit{\mathsf{y}}}_{\mathit{\mathsf{k}}\mathit{\mathsf{i}}}\right| $$

Where *x*_*ti*_ ∈ {0, 1} is the value of gene *i* in model state *s*_*t*_ and *y*_*ki*_ ∈ [0, 1] is the value of gene *i* in data state *v*_*k*_.

The two penalty variables, *ε*_1_ and *ε*_2_, in *g*(*S*, *V*) are used to prevent underfitting and overfitting. *ε*_1_ penalises depending on the proportions of 0 s, *p*_0_, and 1 s, *p*_1_, across all genes and all states in a model state space. The concept of *ε*_1_ is that it penalises complexity in Boolean models by their simulated model state spaces. We have shown that as a Boolean model becomes more complex (i.e. increase in the number of edges), both *p*_0_ and *p*_1_ of its model state space will become closer to 0.5 (See Additional file 5: Figure S4), therefore making *ε*_1_ a good penalty for model complexity.

$$ {\varepsilon}_{\mathsf{1}} = {\mathit{\mathsf{e}}}^{-\mathit{\mathsf{a}}},\ \mathit{\mathsf{where}}\kern0.5em \mathit{\mathsf{a}}={\displaystyle \sum_{\mathit{\mathsf{i}}\in \left\{\mathsf{0},\ \mathsf{1}\right\}}}\frac{{\left({\mathit{\mathsf{p}}}_{\mathit{\mathsf{i}}}-\mathsf{0.5}\right)}^{\mathsf{2}}}{\mathsf{0}.\mathsf{5}} $$

*ε*_2_ penalises based on the number of input genes present in each of the update function *f*_*i*_ in a Boolean model *B*, given a specified threshold *z*_*max*_.

$$ {\varepsilon}_{\mathsf{2}} = {\displaystyle \sum_{\mathit{\mathsf{i}}=\mathsf{1}}^{\mathit{\mathsf{n}}}}{\mathit{\mathsf{w}}}_{\mathit{\mathsf{i}}} $$

Where *w*_*i*_ the penalty for each update function *f*_*i*_ is given by:

$$ {\mathit{\mathsf{w}}}_{\mathit{\mathsf{i}}} = \left\{\begin{array}{ll}\ \frac{{\mathit{\mathsf{z}}}_{\mathit{\mathsf{i}}}-{\mathit{\mathsf{z}}}_{\mathit{\mathsf{max}}}}{\mathit{\mathsf{n}}}, \hfill & \mathit{\mathsf{i}\mathsf{f}}\ {\mathit{\mathsf{z}}}_{\mathit{\mathsf{i}}}>{\mathit{\mathsf{z}}}_{\mathit{\mathsf{max}}}\hfill \\ {}\mathsf{0}, \hfill & \mathit{\mathsf{i}\mathsf{f}}\ {\mathit{\mathsf{z}}}_{\mathit{\mathsf{i}}}\le {\mathit{\mathsf{z}}}_{\mathit{\mathsf{max}}}\hfill \end{array}\right. $$

Where *z*_*i*_ = the number of input genes in update function *f*_*i*_, *z*_*max*_ = the maximum number of input genes allowed per update function. The default *z*_*max*_ in BTR is 6, which means that each target gene is encouraged to have not more than 6 input genes.

#### Search strategy in BTR

**Fig. 9:**
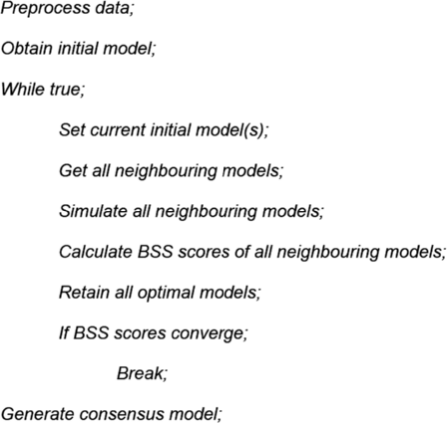
Pseudocode of the search algorithm in BTR

A good search strategy is required in optimisation to locate the optimal solutions within a high dimensional and complex solution space. The search strategy in BTR is a form of swarming hill climbing strategy, in which multiple optimal solutions are kept at each search step and the search only ends when the score converges for all of the optimal solutions (Fig. 9). In BTR search algorithm, the search starts from an initial Boolean model, and iteratively explores the neighbourhood of the current Boolean model in the solution space by minimal modification. When no initial model is given to BTR, it will generate a random initial model whose degree distribution satisfies a power-law distribution with a degree exponent *γ* = 3.

The minimal modification of a Boolean model is performed by adding or removing a gene from a single update function in the Boolean model. The resulting modified model is then evaluated by the BSS scoring function. By repeating this procedure, BTR is able to explore the solution space and eventually arrives at a more optimal Boolean model. Due to the nature of Boolean models that multiple possible Boolean models can give rise to the exact same simulated state space, BTR usually retains a list of equally optimal Boolean models at the end of the search process. In such cases, a consensus model, whose edges are weighted according to the frequencies of their presence in the list of optimal Boolean models, will be generated. Due to the design of the search strategy, it is more geared towards a local search rather than a global search. Therefore in line with the results shown in Fig. 5, BTR is best used for iteratively improving a gene network with known biological knowledge using an independent set of single-cell expression data.

#### BTR data processing

BTR is capable of handling all types of expression data, including qPCR and RNA-Seq. Expression data should be processed and normalised before being used in BTR. In BTR, the expression data is further processed in order to facilitate score calculation by the BSS scoring function. Firstly, if the input data is qPCR expression data, it should be inversed such that the gene with a low expression level should have a low value and vice versa. Finally, the expression values for each gene in the data are scaled to continuous values with a range of 0 ≤ *x* ≤ 1.

### Calculation of F-score

F-score, which is the harmonic average of precision and recall, represents precision and recall concisely [35], is often used to assess the performance of network inference algorithms. Precision denotes the proportion of edges that are truly present among all edges classified as present, while recall denotes the proportion of edges that are truly present among all correctly classified edges (including both edges that are present and absent) [42]. The calculations were performed on directed adjacency matrix.

Precision is defined as:

$$ \mathit{\mathsf{p}}=\frac{\mathit{\mathsf{T}}\mathit{\mathsf{P}}}{\mathit{\mathsf{T}}\mathit{\mathsf{P}}+\mathit{\mathsf{F}}\mathit{\mathsf{P}}} $$

Where *TP* = true positive and *FP* = false positive.

Recall is defined as:

$$ \mathit{\mathsf{r}}=\frac{\mathit{\mathsf{T}}\mathit{\mathsf{P}}}{\mathit{\mathsf{T}}\mathit{\mathsf{P}}+\mathit{\mathsf{F}}\mathit{\mathsf{N}}} $$

Where *TP* = true positive and *FN* = false negative.

F-score is defined as:

$$ \mathit{\mathsf{F}}=\frac{\mathsf{2}\mathit{\mathsf{p}}\mathit{\mathsf{r}}}{\mathit{\mathsf{r}}+\mathit{\mathsf{p}}} $$

### Synthetic data

The synthetic data used for comparing scoring functions and network inference algorithms consist of true networks, expression data and lists of modified networks. The true networks and expression data were generated using GeneNetWeaver version 3.13 [28]. The true networks contain 10 genes each and were extracted from the gene network of yeast. Each true network generated by GeneNetWeaver was then categorised into acyclic and cyclic networks. A total of 5 acyclic and 5 cyclic true networks were used in this study. The expression data were generated using ordinary and stochastic differential equations based on the true networks. A single time series expression data with 1000 observations were generated per true network, and the expression data were simulated under steady state wild type condition. A coefficient of 0.05 was used for noise term in the stochastic differential equations. The synthetic expression data as generated by GeneNetWeaver is used as non zero-inflated data. In addition, the synthetic expression data is converted into a zero-inflated data to simulate drop-outs in single-cell expression data by calculating the probability of a reading being a drop-out (i.e. zero value) based on its expression level. The probability of a reading being a drop-out, *p*_*d*_, is modelled using the following equation:

$$ {\mathit{\mathsf{p}}}_{\mathit{\mathsf{d}}} = {\mathsf{2}}^{-\mathit{\mathsf{c}}\mathit{\mathsf{y}}} $$

Where *c* = a constant (in this study, *c* = 6), and *y* = a reading of the expression level of a particular gene,

The lists of modified networks were generated in R using the bnlearn package [43] for Bayesian networks and the BTR package for Boolean models. The modified networks were generated by modifying the number of edges that differ from the true network, ranging from 2 edges up to 40 differing edges. The modified Bayesian networks and the modified Boolean models were generated separately due to different underlying structural constraints imposed by each framework. In Bayesian framework all networks must be directed acyclic graphs, while Boolean models do not have such restrictions. In contrast, Boolean models require explicit specification of activation and inhibition edges, while Bayesian networks handle activation and inhibition implicitly without modifying the edges. Although the generation of modified Bayesian networks and Boolean models were done separately and therefore they are not identical, all modified networks contain the same number of differing edges (2 to 40 edges) with respect to the true network. Note that the differences in edges for acyclic modified networks are not cumulative, due to difficulties in generating a directed acyclic graph with cumulative edge differences. The differences in edges for cyclic modified networks are also not cumulative to maintain consistency with the acyclic modified networks.

For synthetic data, the initial state used for the simulation of Boolean models is the expression values at time *t* = 0.

### Haematopoietic data

Two Boolean models of haematopoiesis were used as initial models for model learning in this study, namely Krumsiek [39] and Bonzanni models [38]. The update functions of both models were converted into functions with an activation clause and an inhibition clause, in which each of the clauses are individually expressed in disjunctive normal form. Note that one of the nodes (EgrNab) in the Krumsiek model comprises of 3 different genes, Egr-1, Egr-2 and Nab-2. The initial states used in the simulation were obtained from both papers respectively.

A single-cell qPCR data and a single-cell RNA-Seq data, both obtained from Wilson et al. [10], were used for model learning. The single-cell qPCR data contain 44 genes from 1626 cells (992 HSCs, 178 LMPPs, 147 CMPs, 185 GMPs and 124 MEPs), while the single-cell RNA-Seq data are collected from 96 HSCs. The expression data are processed and normalised as described in the original paper.

For Bonzanni and Krumsiek models, the initial states used for the simulation Boolean models are obtained from each paper respectively.

### Network inference algorithms and analyses software used

BIC and its associated hill-climbing algorithm are implemented in bnlearn [43]. BestFit [29] is an algorithm for inferring Boolean models under synchronous framework implemented in BoolNet [44]. ARACNE [30] and CLR [31] are inference algorithms for inferring relevance networks based on mutual information. bc3net [32] and GeneNet [33] are inference algorithms based on Bayesian networks, while GENIE3 is a type of tree-based methods [34].

Plots in this study were generated using ggplot2 [45], except network plots that were generated using Cytoscape [46] and heat maps that were generated using gplots [47]. Steady state analysis was performed using genYsis [48], which search for steady states reachable from all possible initial states.

## Additional files

Additional file 1: Figure S1. Is a PowerPoint file containing the results of comparing BIC and BSS scoring functions with acyclic networks using non zero-inflated synthetic expression data. (PPTX 491 kb) Additional file 2: Figure S2. Is a PowerPoint file containing the results of comparing BIC and BSS scoring functions with cyclic networks using non zero-inflated synthetic expression data. (PPTX 366 kb) Additional file 3: Figure S3. Is a PowerPoint file containing the summary results for both BIC and BSS scoring functions across all networks using non zero-inflated synthetic expression data. (PPTX 213 kb) Additional file 4: Figure S5. Is a PowerPoint file containing detailed update functions of Boolean models discussed in this study. (PPTX 587 kb) Additional file 5: Figure S4. Is a PowerPoint file containing a plot that explains and justifies the use of *ε*_1_ as a penalty variable in BSS scoring function. (PPTX 124 kb)
