# Supplementary material for: BTR: training asynchronous Boolean models using single-cell expression data
Source: BMC Bioinformatics. 2016 Sep 6;17(1):355. doi: 10.1186/s12859-016-1235-y (PMC5012073; doi:10.1186/s12859-016-1235-y)
Supplement: Additional file 1: Figure S1. — Is a PowerPoint file containing the results of comparing BIC and BSS scoring functions with acyclic networks using non zero-inflated synthetic expression data. (PPTX 491 kb) [file 12859_2016_1235_MOESM1_ESM.pptx]

## Slide 1
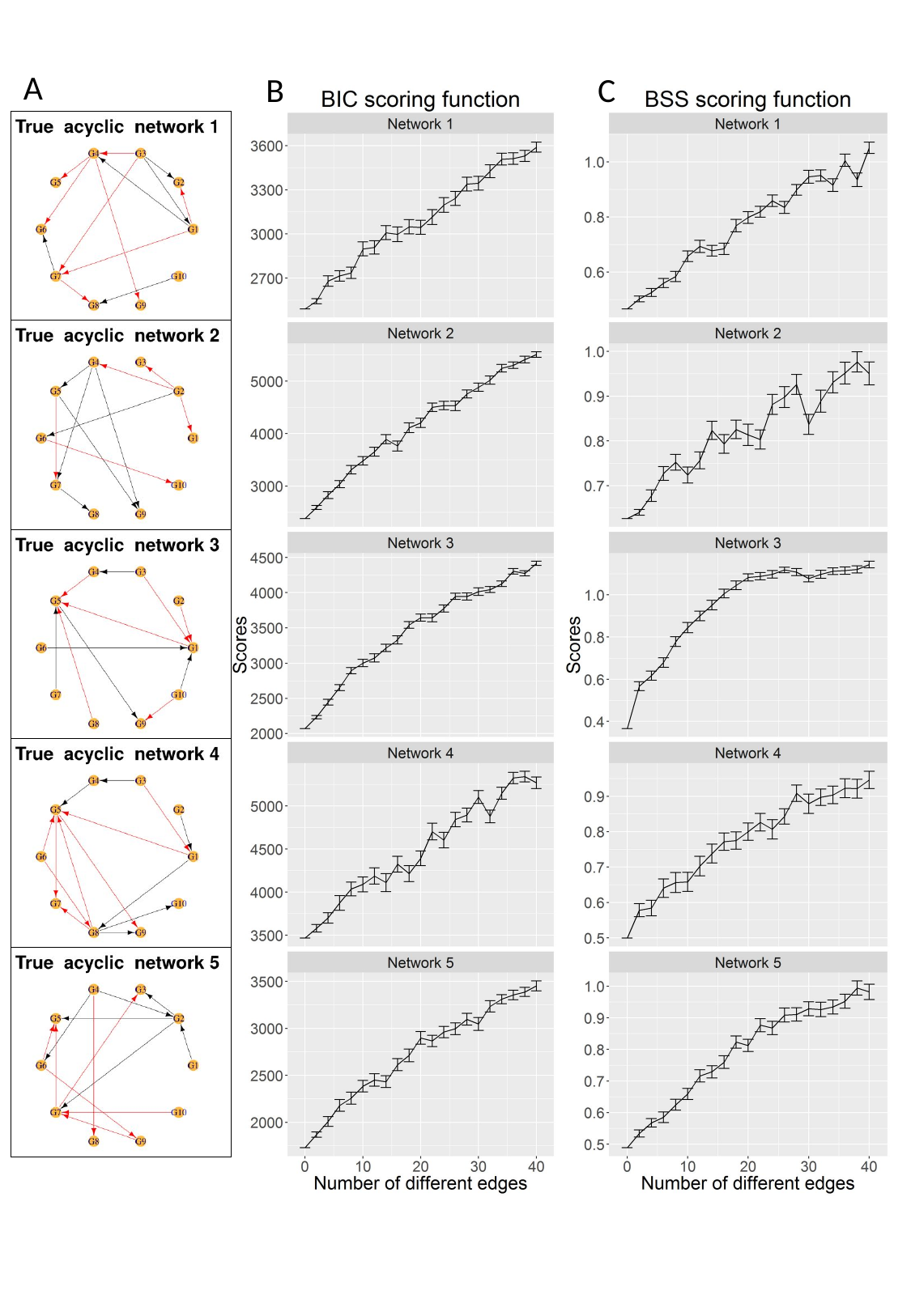

A
B
C

## Slide 2
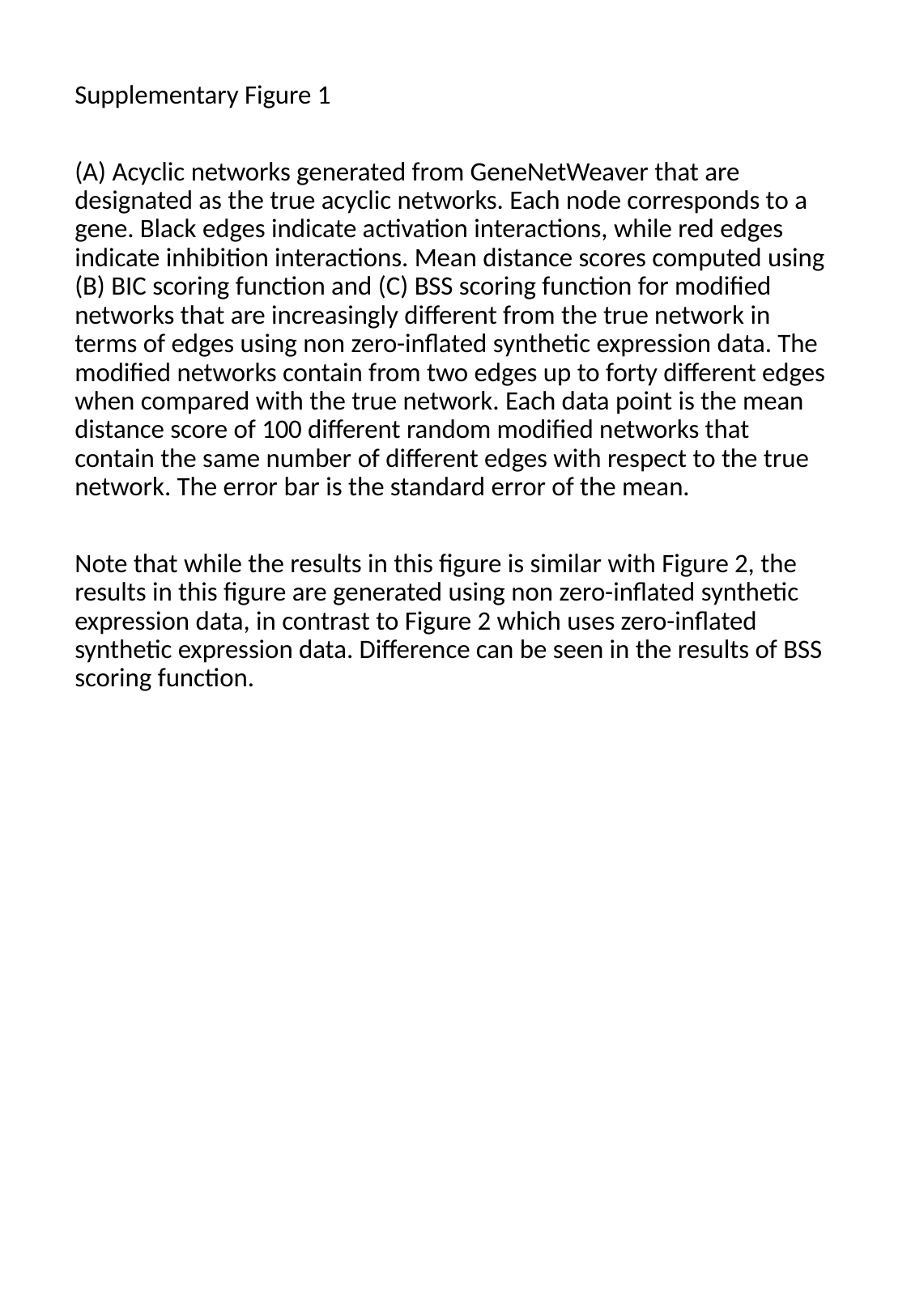

Supplementary Figure 1
(A) Acyclic networks generated from GeneNetWeaver that are designated as the true acyclic networks. Each node corresponds to a gene. Black edges indicate activation interactions, while red edges indicate inhibition interactions. Mean distance scores computed using (B) BIC scoring function and (C) BSS scoring function for modified networks that are increasingly different from the true network in terms of edges using non zero-inflated synthetic expression data. The modified networks contain from two edges up to forty different edges when compared with the true network. Each data point is the mean distance score of 100 different random modified networks that contain the same number of different edges with respect to the true network. The error bar is the standard error of the mean.
Note that while the results in this figure is similar with Figure 2, the results in this figure are generated using non zero-inflated synthetic expression data, in contrast to Figure 2 which uses zero-inflated synthetic expression data. Difference can be seen in the results of BSS scoring function.
