# Supplementary material for: BTR: training asynchronous Boolean models using single-cell expression data
Source: BMC Bioinformatics. 2016 Sep 6;17(1):355. doi: 10.1186/s12859-016-1235-y (PMC5012073; doi:10.1186/s12859-016-1235-y)
Supplement: Additional file 3: Figure S3. — Is a PowerPoint file containing the summary results for both BIC and BSS scoring functions across all networks using non zero-inflated synthetic expression data. (PPTX 213 kb) [file 12859_2016_1235_MOESM3_ESM.pptx]

## Slide 1
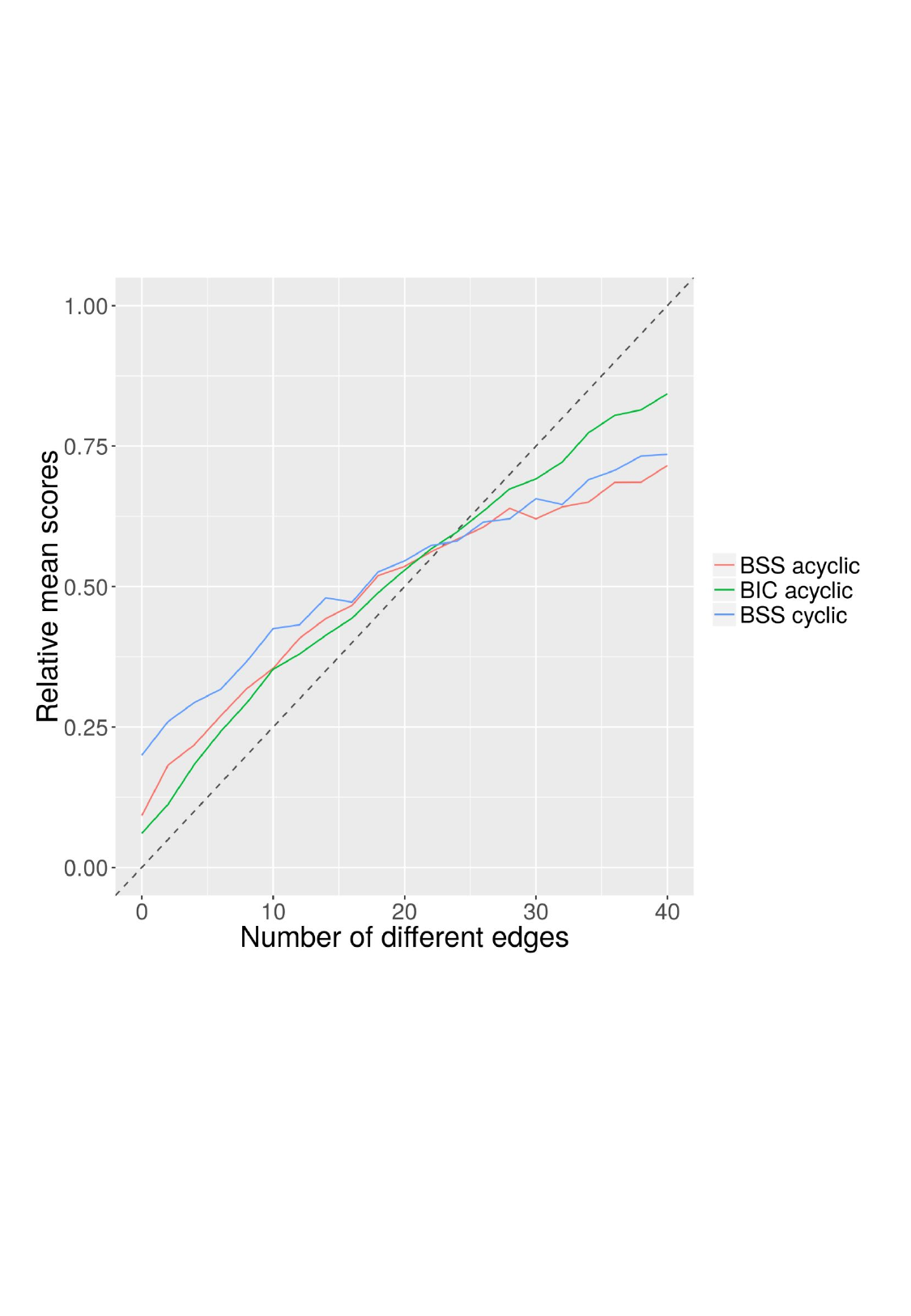

## Slide 2
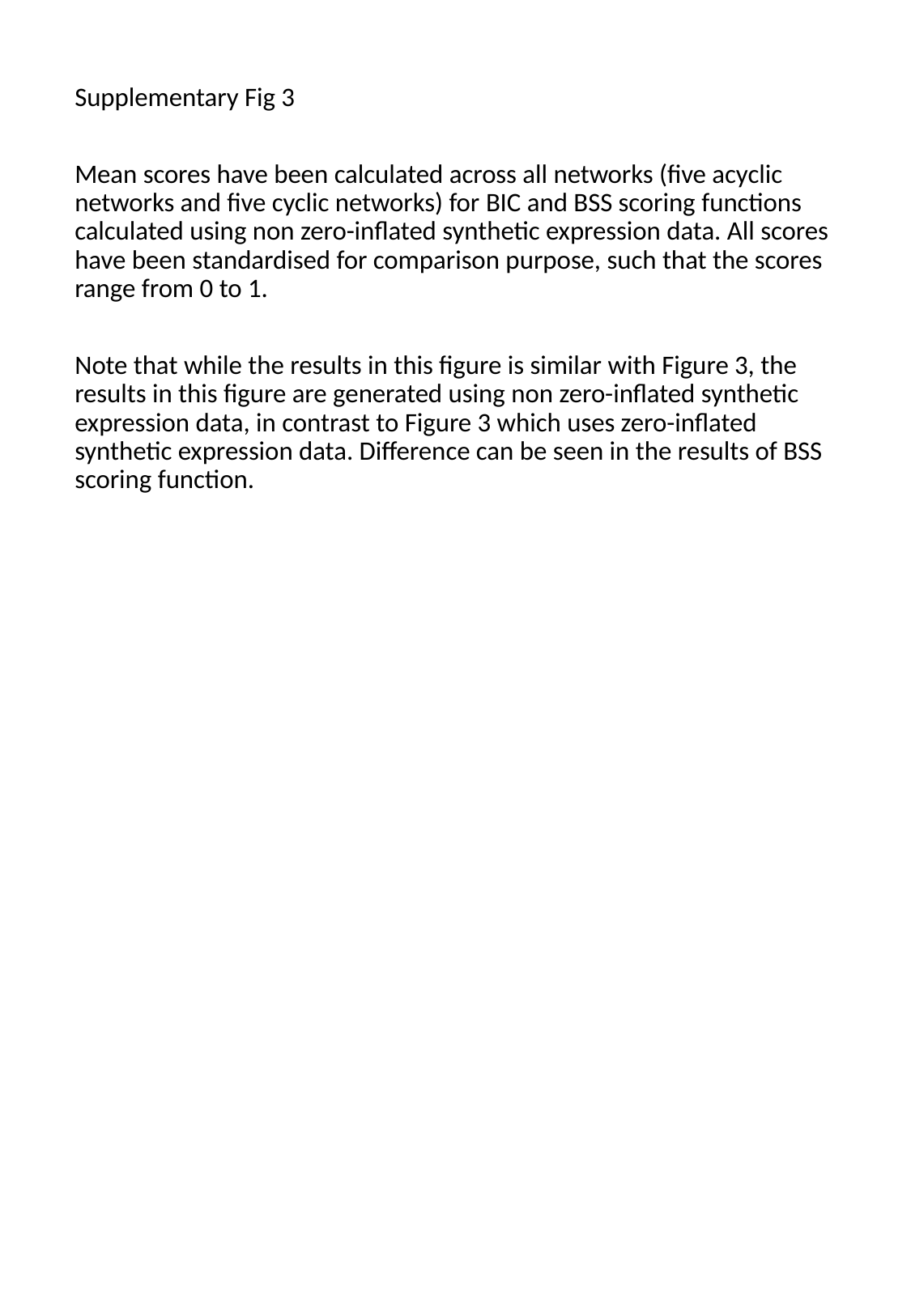

Supplementary Fig 3
Mean scores have been calculated across all networks (five acyclic networks and five cyclic networks) for BIC and BSS scoring functions calculated using non zero-inflated synthetic expression data. All scores have been standardised for comparison purpose, such that the scores range from 0 to 1.
Note that while the results in this figure is similar with Figure 3, the results in this figure are generated using non zero-inflated synthetic expression data, in contrast to Figure 3 which uses zero-inflated synthetic expression data. Difference can be seen in the results of BSS scoring function.
