# Supplementary material for: BTR: training asynchronous Boolean models using single-cell expression data
Source: BMC Bioinformatics. 2016 Sep 6;17(1):355. doi: 10.1186/s12859-016-1235-y (PMC5012073; doi:10.1186/s12859-016-1235-y)
Supplement: Additional file 4: Figure S5. — Is a PowerPoint file containing detailed update functions of Boolean models discussed in this study. (PPTX 587 kb) [file 12859_2016_1235_MOESM4_ESM.pptx]

## Slide 1
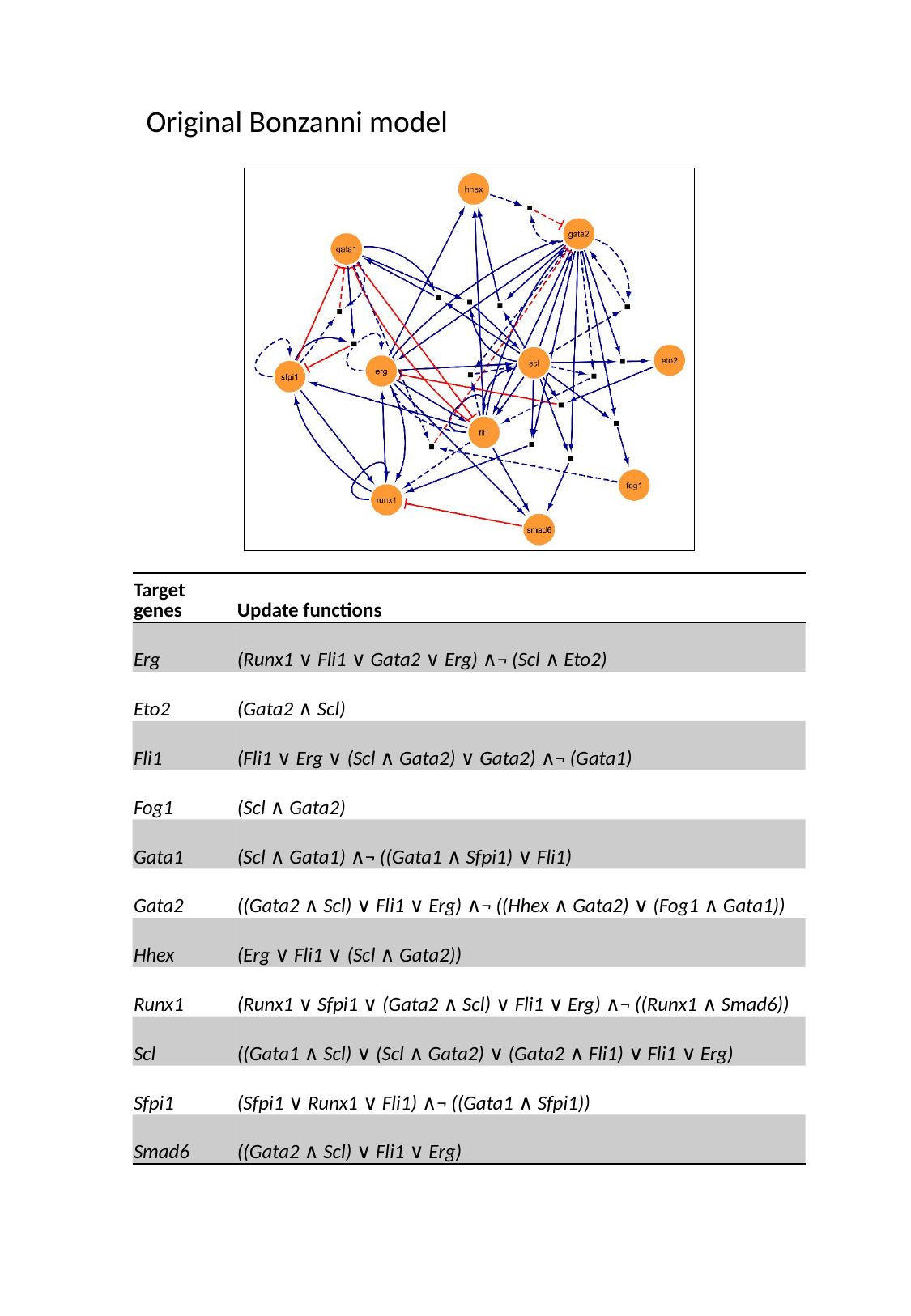

Original Bonzanni model
| Target genes | Update functions |
| --- | --- |
| Erg | (Runx1 ∨ Fli1 ∨ Gata2 ∨ Erg) ∧¬ (Scl ∧ Eto2) |
| Eto2 | (Gata2 ∧ Scl) |
| Fli1 | (Fli1 ∨ Erg ∨ (Scl ∧ Gata2) ∨ Gata2) ∧¬ (Gata1) |
| Fog1 | (Scl ∧ Gata2) |
| Gata1 | (Scl ∧ Gata1) ∧¬ ((Gata1 ∧ Sfpi1) ∨ Fli1) |
| Gata2 | ((Gata2 ∧ Scl) ∨ Fli1 ∨ Erg) ∧¬ ((Hhex ∧ Gata2) ∨ (Fog1 ∧ Gata1)) |
| Hhex | (Erg ∨ Fli1 ∨ (Scl ∧ Gata2)) |
| Runx1 | (Runx1 ∨ Sfpi1 ∨ (Gata2 ∧ Scl) ∨ Fli1 ∨ Erg) ∧¬ ((Runx1 ∧ Smad6)) |
| Scl | ((Gata1 ∧ Scl) ∨ (Scl ∧ Gata2) ∨ (Gata2 ∧ Fli1) ∨ Fli1 ∨ Erg) |
| Sfpi1 | (Sfpi1 ∨ Runx1 ∨ Fli1) ∧¬ ((Gata1 ∧ Sfpi1)) |
| Smad6 | ((Gata2 ∧ Scl) ∨ Fli1 ∨ Erg) |

## Slide 2
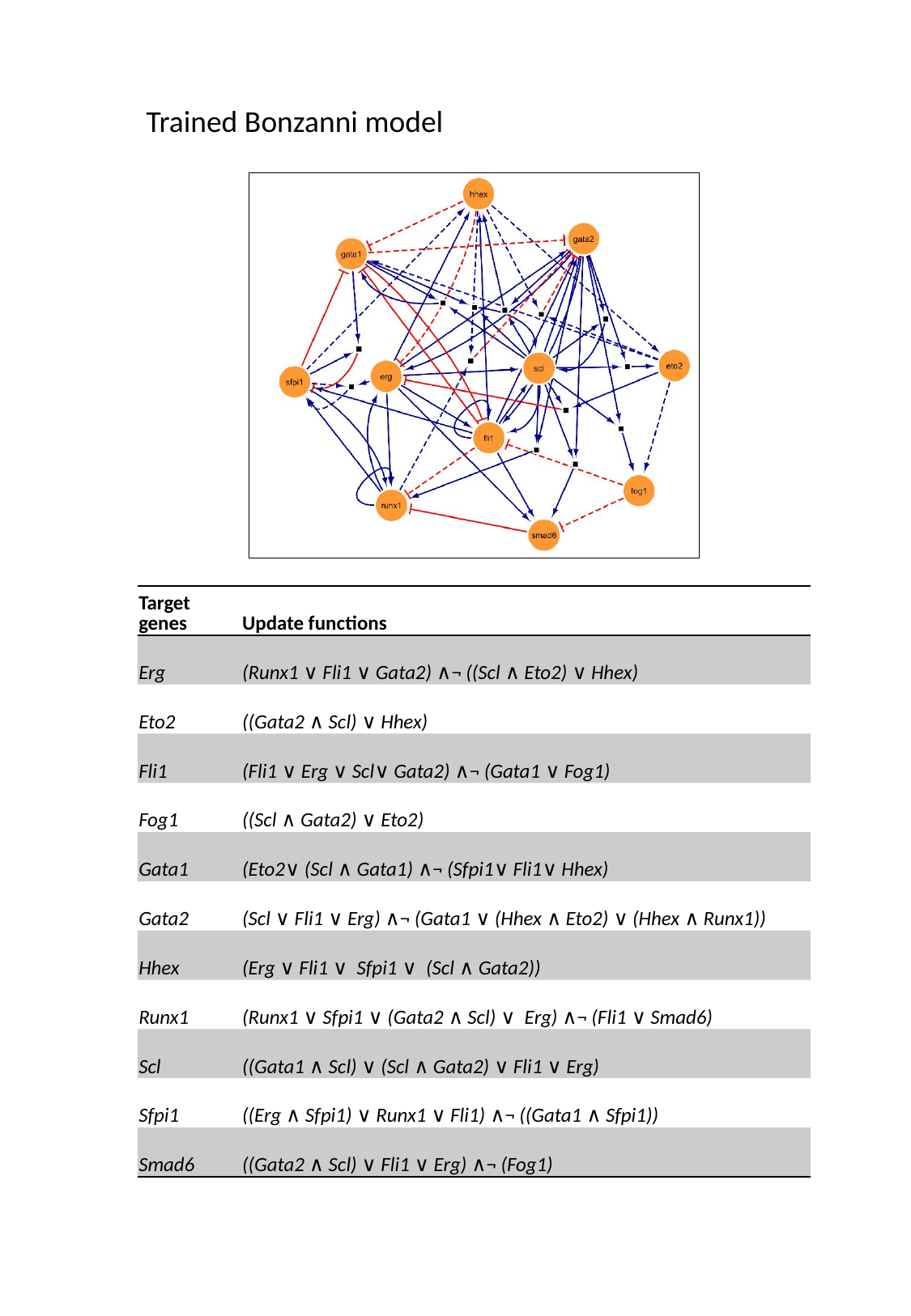

Trained Bonzanni model
| Target genes | Update functions |
| --- | --- |
| Erg | (Runx1 ∨ Fli1 ∨ Gata2) ∧¬ ((Scl ∧ Eto2) ∨ Hhex) |
| Eto2 | ((Gata2 ∧ Scl) ∨ Hhex) |
| Fli1 | (Fli1 ∨ Erg ∨ Scl∨ Gata2) ∧¬ (Gata1 ∨ Fog1) |
| Fog1 | ((Scl ∧ Gata2) ∨ Eto2) |
| Gata1 | (Eto2∨ (Scl ∧ Gata1) ∧¬ (Sfpi1∨ Fli1∨ Hhex) |
| Gata2 | (Scl ∨ Fli1 ∨ Erg) ∧¬ (Gata1 ∨ (Hhex ∧ Eto2) ∨ (Hhex ∧ Runx1)) |
| Hhex | (Erg ∨ Fli1 ∨ Sfpi1 ∨ (Scl ∧ Gata2)) |
| Runx1 | (Runx1 ∨ Sfpi1 ∨ (Gata2 ∧ Scl) ∨ Erg) ∧¬ (Fli1 ∨ Smad6) |
| Scl | ((Gata1 ∧ Scl) ∨ (Scl ∧ Gata2) ∨ Fli1 ∨ Erg) |
| Sfpi1 | ((Erg ∧ Sfpi1) ∨ Runx1 ∨ Fli1) ∧¬ ((Gata1 ∧ Sfpi1)) |
| Smad6 | ((Gata2 ∧ Scl) ∨ Fli1 ∨ Erg) ∧¬ (Fog1) |

## Slide 3
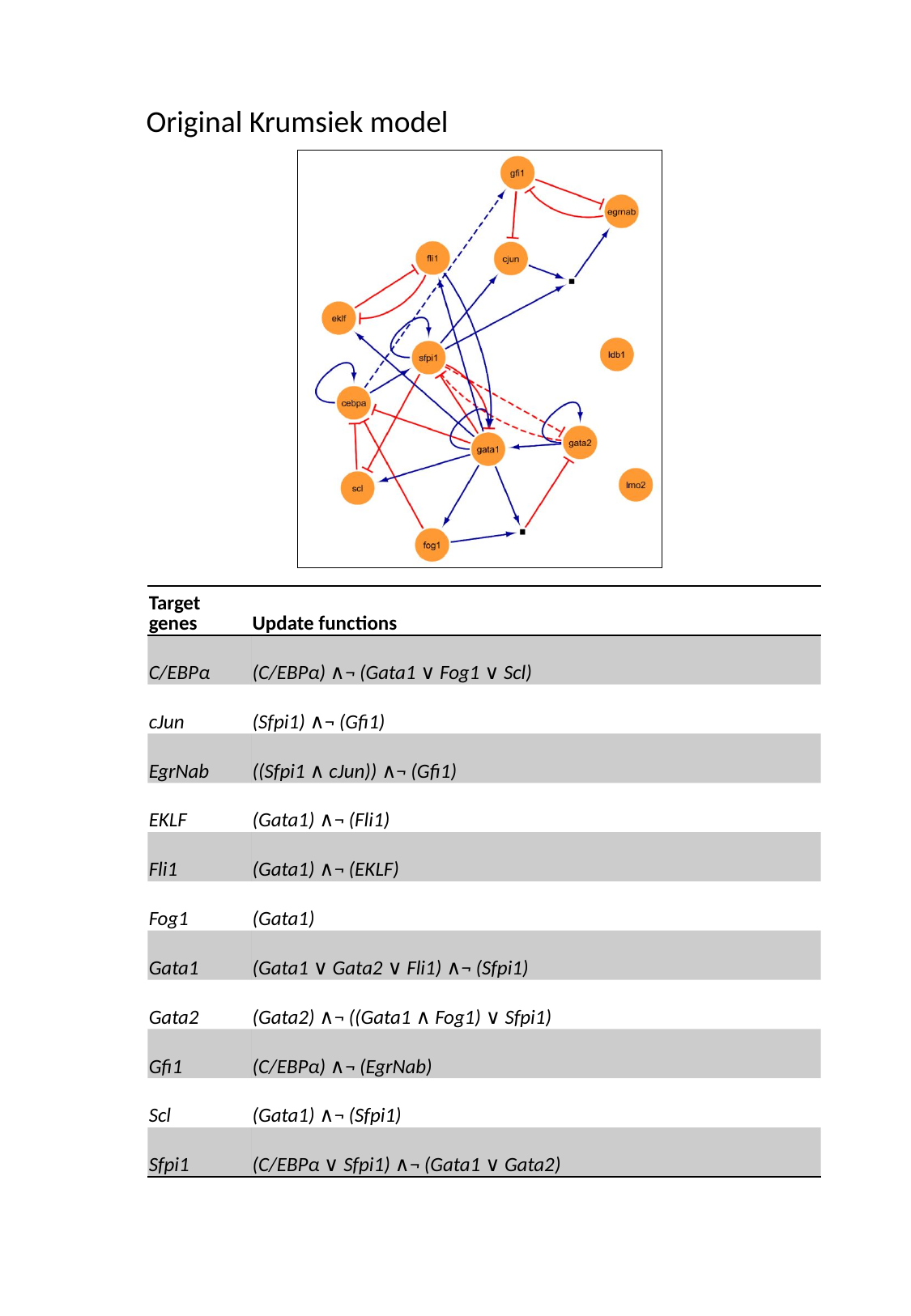

Original Krumsiek model
| Target genes | Update functions |
| --- | --- |
| C/EBPα | (C/EBPα) ∧¬ (Gata1 ∨ Fog1 ∨ Scl) |
| cJun | (Sfpi1) ∧¬ (Gfi1) |
| EgrNab | ((Sfpi1 ∧ cJun)) ∧¬ (Gfi1) |
| EKLF | (Gata1) ∧¬ (Fli1) |
| Fli1 | (Gata1) ∧¬ (EKLF) |
| Fog1 | (Gata1) |
| Gata1 | (Gata1 ∨ Gata2 ∨ Fli1) ∧¬ (Sfpi1) |
| Gata2 | (Gata2) ∧¬ ((Gata1 ∧ Fog1) ∨ Sfpi1) |
| Gfi1 | (C/EBPα) ∧¬ (EgrNab) |
| Scl | (Gata1) ∧¬ (Sfpi1) |
| Sfpi1 | (C/EBPα ∨ Sfpi1) ∧¬ (Gata1 ∨ Gata2) |

## Slide 4
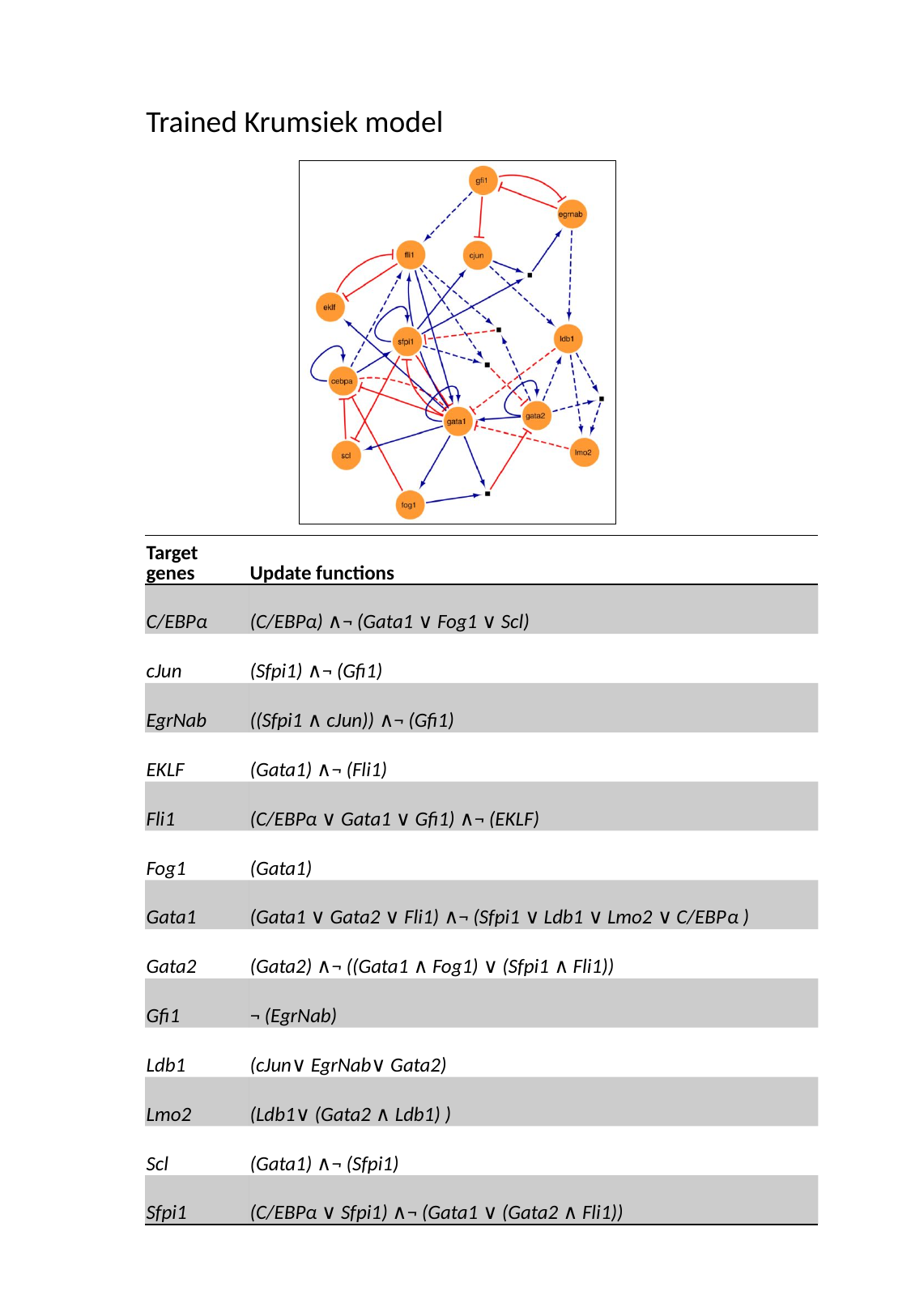

Trained Krumsiek model
| Target genes | Update functions |
| --- | --- |
| C/EBPα | (C/EBPα) ∧¬ (Gata1 ∨ Fog1 ∨ Scl) |
| cJun | (Sfpi1) ∧¬ (Gfi1) |
| EgrNab | ((Sfpi1 ∧ cJun)) ∧¬ (Gfi1) |
| EKLF | (Gata1) ∧¬ (Fli1) |
| Fli1 | (C/EBPα ∨ Gata1 ∨ Gfi1) ∧¬ (EKLF) |
| Fog1 | (Gata1) |
| Gata1 | (Gata1 ∨ Gata2 ∨ Fli1) ∧¬ (Sfpi1 ∨ Ldb1 ∨ Lmo2 ∨ C/EBPα ) |
| Gata2 | (Gata2) ∧¬ ((Gata1 ∧ Fog1) ∨ (Sfpi1 ∧ Fli1)) |
| Gfi1 | ¬ (EgrNab) |
| Ldb1 | (cJun∨ EgrNab∨ Gata2) |
| Lmo2 | (Ldb1∨ (Gata2 ∧ Ldb1) ) |
| Scl | (Gata1) ∧¬ (Sfpi1) |
| Sfpi1 | (C/EBPα ∨ Sfpi1) ∧¬ (Gata1 ∨ (Gata2 ∧ Fli1)) |
