# Supplementary figures and images for: BTR: training asynchronous Boolean models using single-cell expression data
Source: BMC Bioinformatics. 2016 Sep 6;17(1):355. doi: 10.1186/s12859-016-1235-y (PMC5012073; doi:10.1186/s12859-016-1235-y)

## Slide 1
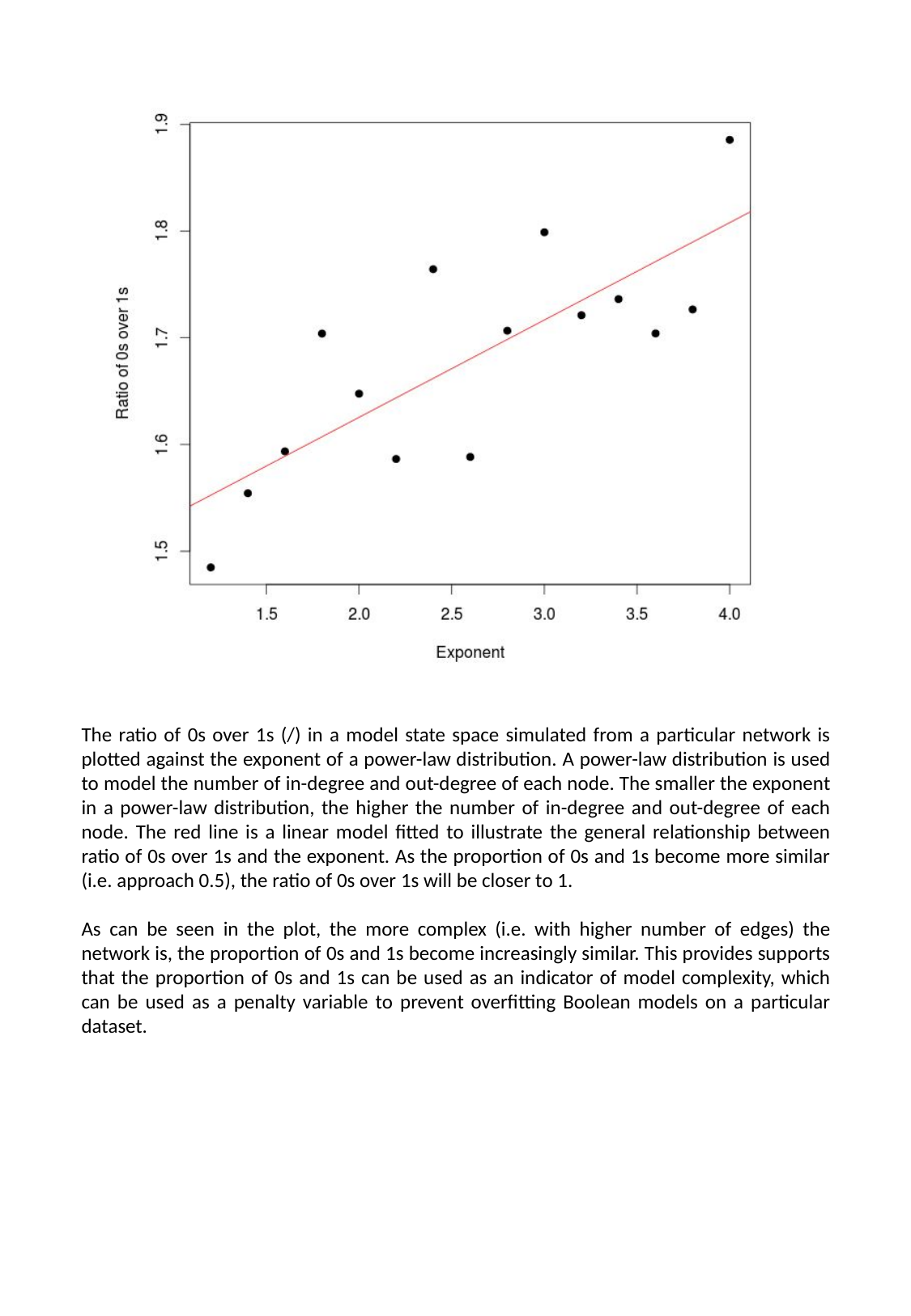

Supplement: Additional file 5: Figure S4. — Is a PowerPoint file containing a plot that explains and justifies the use of ε 1 as a penalty variable in BSS scoring function. (PPTX 124 kb) [file 12859_2016_1235_MOESM5_ESM.pptx]
